# Dynamic QTLs for sugars and enzyme activities provide an overview of genetic control of sugar metabolism during peach fruit development

**DOI:** 10.1093/jxb/erw169

**Published:** 2016-04-25

**Authors:** Elsa Desnoues, Valentina Baldazzi, Michel Génard, Jehan-Baptiste Mauroux, Patrick Lambert, Carole Confolent, Bénédicte Quilot-Turion

**Affiliations:** ^1^Génétique et Amélioration des Fruits et Légumes, INRA, 84000 Avignon, France; ^2^Plantes et Systèmes de Culture Horticoles, INRA, 84000 Avignon, France

**Keywords:** Dynamic QTLs, enzymes, fruit, genetic control, *Prunus persica*, sugar metabolism.

## Abstract

Knowledge of the genetic control of sugar metabolism is essential to enhance fruit quality and promote fruit consumption. The sugar content and composition of fruits varies with species, cultivar and stage of development, and is controlled by multiple enzymes. A QTL (quantitative trait locus) study was performed on peach fruit [*Prunus persica* (L.) Batsch], the model species for *Prunus*. Progeny derived from an interspecific cross between *P. persica* cultivars and *P. davidiana* was used. Dynamic QTLs for fresh weight, sugars, acids, and enzyme activities related to sugar metabolism were detected at different stages during fruit development. Changing effects of alleles during fruit growth were observed, including inversions close to maturity. This QTL analysis was supplemented by the identification of genes annotated on the peach genome as enzymes linked to sugar metabolism or sugar transporters. Several cases of co-locations between annotated genes, QTLs for enzyme activities and QTLs controlling metabolite concentrations were observed and discussed. These co-locations raise hypotheses regarding the functional regulation of sugar metabolism and pave the way for further analyses to enable the identification of the underlying genes. In conclusion, we identified the potential impact on fruit breeding of the modification of QTL effect close to maturity.

## Highlight

Forty-five QTLs controlling sugars and enzyme activities related to sugar metabolism in peach fruit were identified. Dynamic QTLs revealed changing effects of alleles during fruit development.

## Introduction

Because of its relatively short juvenile period, its small genome and the quality of its genome sequence, peach [*Prunus persica* (L.) Batsch] is an ideal model species, at least for *Prunus* (Shulaev *et al.*, 2008; Arús *et al.*, 2012). For the fruit growing industry, the present challenge is to increase fruit consumption, which mainly relies on enhancing taste and nutritional quality. The sugar-to-acid ratio is the criterion that most affects the taste of the fruit (Colaric *et al.*, 2005). Moreover, sugar composition affects sweetness (Schaffer, 1999). In peach, the major sugar at maturity is sucrose, followed by glucose and fructose and then sorbitol. High natural variability of sugar concentrations can be observed between genotypes (Cantín *et al.*, 2009). Sugar metabolism is key because, in addition to controlling sweetness, it feeds respiration and acid metabolism via glycolysis and provides carbon for the synthesis of structural compounds (e.g. cell wall). The sugar metabolism network in fruit is fairly complex, and many enzymes are involved in the interconversion of different sugars. For a complete overview of the sugar metabolism network in peach, see Supplementary Fig. S1 at *JXB* online. The profiles of sugar concentration during fruit growth differ for different sugars (Moriguchi *et al.*, 1990*a*). Such specific time courses suggest specific regulation of the different mechanisms controlling sugar metabolism during fruit growth. Studying the profiles of enzyme capacities (maximum activities) not only completes the picture of sugar metabolism but also may help to identify key steps of regulation (Moriguchi *et al.*, 1990*b*; Kanayama *et al.*, 2005) during fruit development and among different genotypes (Desnoues *et al.*, 2014).

Knowledge of the gene architecture involved in sugar metabolism can provide further insight into the mechanisms underlying the variations in sugar concentrations. Studies from different species highlight a complex and quantitative genetic determinism of sugars (Labate *et al.*, 2007; Lerceteau-Köhler *et al.*, 2012). In peach, specific loci responsible for variations in sugar concentration of the fruit have been identified in each of the eight linkage groups (Sosinski *et al.*, 1998; Dirlewanger *et al.*, 1999; Etienne *et al.*, 2002; Quilot *et al.*, 2004). Several co-locations were observed between QTLs (quantitative trait loci) linked to different sugars or related to other quality traits. Opposite allelic effects on sugar concentration and fruit weight observed at some common loci (Grandillo *et al.*, 1999) suggest pleiotropic effects. For example, a single gene was found to have pleiotropic effects on wheat grain protein and zinc and iron concentrations (Uauy *et al.*, 2006). However, the genes responsible for the QTL detected remain unknown in most cases. Thus, it is difficult to know if those co-locations are due to the pleiotropic effects of a gene or to the action of several genes linked in clusters.

High variations in sugar concentrations at maturity, as in peach (Cantín *et al.*, 2009), may result from differential changes of sugar content during growth. However, in peach, all QTLs related to sugar metabolism in fruit identified so far referred to mature fruit only. Dynamic QTLs would highlight the complex series of genetically programmed events leading to quality at maturity. Sun *et al.* (2012) showed that the concentration of lycopene in tomato is under complex genetic control with several loci involved at different stages of development. Studying the change of apple firmness and softening after harvest, Costa *et al.* (2010) identified three novel genomic regions influencing various physiological aspects of texture. To date, no study has attempted to identify loci involved in the time course of sugar metabolism during fruit growth.

Dynamic QTLs for enzyme capacities may aid in the understanding of the mechanisms controlling variations in metabolites. Indeed, co-locations between QTLs for enzyme capacity and a related metabolite strongly indicate functional links. In maize, several loci have been identified that are associated with both variations in enzyme capacities and sugar concentrations and thereby clarify the metabolic pathways involved in the variation of some metabolites (Causse *et al.*, 1994; Thévenot *et al.*, 2005). Some studies have identified co-locations between candidate genes coding for enzymes and QTLs responsible for the variability in peach fruit quality (Ogundiwin *et al.*, 2009; Illa *et al.*, 2011). The annotation of the peach genome (The International Peach Genome Initiative, 2013) offers new possibilities for the study of candidate genes and provides essential assistance in understanding the mechanisms involved in sugar metabolism.

The objective of this study is to dissect the genetic control of sugar metabolism in peach fruit. For this, we mapped QTLs in peach progeny derived from an interspecific cross. QTLs for sugars, acids and related enzymatic capacities were identified at different stages during fruit growth. The stability of the QTLs across developmental stages and the co-locations between QTLs were specifically investigated to draw functional hypotheses. In addition, we searched for candidate genes related to sugar metabolism based on the annotation of the peach genome. The co-locations of these genes and QTLs were examined to identify candidates responsible for the natural variability of sugar concentration. Finally, we discussed the implications of these findings for breeding programmes.

## Materials and methods

### Plant materials

The breeding population was derived from an interspecific cross between *Prunus persica* and a wild close relative, clone P1908 of *P. davidiana* (Pascal *et al.*, 1998). P1908 (D) with small green fruit was crossed with *P. persica* ‘Summergrand’ (S), and an F1 progeny (SD) was obtained. One F1 hybrid was then back-crossed to S to produce a BC1 progeny. Finally, BC1 individuals were used to pollinate *P. persica* ‘Zephyr’ (Z) to derive the breeding population (BC2). S and Z are yellow and white nectarine cultivars, respectively, with large tasty fruits. For brevity and clarity, this population will be referred to as BC2 throughout this manuscript, although the *P. persica* parents (P) used to produce the BC1 and BC2 progeny are not identical. The possible genotypes at any given locus in the BC2 progeny are presented in Table 1.

**Table 1.** Possible genotypes at a single locus in SD, BC1 and BC2 progenies (from Quilot et al., 2004)

D x S			SD40 x S			BC1 x Z			
**SD**	D_1_	D_2_	**BC1**	D_1_	S_1_	**BC2**	D_1_	S_1_	S_2_
S_1_	D_1_S_1_	D_2_S_1_	S_1_	D_1_S_1_	S_1_S_1_	Z_1_	Z_1_D_1_ (1/8)	Z_1_S_1_ (1/4)	Z_1_S_2_ (1/8)
S_2_	D_1_S_2_	D_2_S_2_	S_2_	D_1_S_2_	S_1_S_2_	Z_2_	Z_2_D_1_ (1/8)	Z_2_S_1_ (1/4)	Z_2_S_2_ (1/8)
SD40 genotype is coded D1S1 at one locus			Possible gametes from BC1 progeny						
			D_1_ (1/4)	S_1_ (1/2)	S_2_ (1/4)				

One tree per genotype was planted in a randomized design in the orchard of the INRA Research Centre of Avignon (southern France). Trees were three years old when planted in the orchard, in 2001. All of the genotypes were grafted on GF305 seedling rootstocks and were grown under normal irrigation, fertilization and pest control conditions. All of the trees were homogeneously pruned and thinned.

### Phenotyping

In 2012, six fruits per genotype were collected on each sampling date for 106 genotypes of the progeny described above. They were weighed and peeled, and the mesocarp was cut into small pieces and pooled to give a single sample. Samples were immediately snap frozen in liquid nitrogen and stored at −80 °C. The frozen samples were then ground to a fine powder in liquid nitrogen and stored at −80 °C for future analyses. Two technical replicates were performed for each sample. The technical replicates correspond to two distinct extractions and assays. For the metabolite and enzymatic assays, 20mg aliquots of powdered mesocarp were extracted as described in Gibon *et al.* (2009).

Nineteen phenotypic traits were measured in the samples: fresh weight (FW); concentrations of sucrose (Suc), sorbitol (Sor), fructose (Fru), glucose (Glc), malate (Mal), and citrate (Cit); and enzyme capacities for sucrose synthase (SuSy, EC 2.4.1.13), neutral invertase (NI, EC 3.2.1.26), acid invertase (AI, EC 3.2.1.26), sorbitol dehydrogenase (SDH, EC 1.1.1.14), sorbitol oxidase (SO), fructokinase (FK, EC 2.7.1.4), hexokinase (HK, EC 2.7.1.1), ATP-phosphofructokinase (PFK, EC 2.7.1.11), fructose-1,6-bisphosphatase (F1,6BPase, EC 3.1.3.11), phosphoglucomutase (PGM, EC 5.4.2.2), UDP-glucose pyrophosphorylase (UGPase, EC 2.7.7.9), and sucrose phosphate synthase (SPS, EC 2.4.1.14). These assays, presented by Desnoues *et al.* (2014) with the exception of acid concentration, were performed at saturating concentration of all substrates. Following the same sample preparation and extraction method as for the sugar assay presented in Desnoues *et al.* (2014), malate concentrations were measured as described by Gibon *et al.* (2009), and citrate concentrations were measured as described by Moellering and Gruber (1966).

Knowing the approximate maturity dates of each genotype (data from previous years), we forecasted six sampling dates for each genotype during fruit development corresponding to approximately 40, 52, 64, 76, 88 and 100% of the length of development. However, as the maturity date strongly depends on environmental conditions, the actual maturity date was different from the one estimated a priori. As a result, the sampling dates did not correspond to the same percentage of development for all genotypes. For this reason we then rescaled the phenotyping data. For all genotypes and traits, a fit by local regression was performed with the loess function (Cleveland *et al.*, 1991) using R software (R Development Core Team, 2011). As the data were slightly erratic for some traits, two adjustments were applied consecutively to maintain both the order of magnitude and the general trend of the data. Rescaled stages were defined considering the percentage of total fruit development duration as the time unit. To reproduce the same design as the experimental data, the total fruit development duration was divided into five equal parts starting at 40%, which corresponds to the earlier percentage common to all genotypes. Six values were thus extracted from the fitted curves at 40, 52, 64, 76, 88 and 100% of fruit development for all genotypes and traits. See Supplementary Fig. S2 for an example.

### DNA isolation and SNP genotyping

Genotyping of the BC2 population was performed using the International Peach SNP Consortium (IPSC) 9K peach SNP array v1 (Illumina Inc. San Diego, CA, USA), which was described in Verde *et al.* (2012).

For SNP array genotyping, isolation of genomic DNA and subsequent Infinium II assays were performed as explained in Verde *et al.* (2012). DNA was extracted with the DNeasy 96 Plant kit (Qiagen, MD, USA), diluted to 50ng μl^−1^ and sent to the IASMA Research and Innovation Centre (San Michele all’Adige, Italy) for genotyping. The assays were performed following the manufacturer’s recommendations. SNP genotypes were scored with the Genotyping Module of GenomeStudio Data Analysis software (Illumina Inc. San Diego, CA, USA), using a GenCall threshold of 0.15. SNPs with GenTrain scores <0.6 were used to clean up the data file. SNPs showing severe segregation distortion (χ^2^ test, *P*<10^−6^) and more than 1% of missing data were excluded.

### Genetic linkage map construction

In this study, two different genetic linkage maps derived from the BC2 progeny were used to perform the QTL analyses: one monitoring the polymorphisms between the D and S genomes and the second tracking the heterozygosity of Z. They will hereafter be referred to as the ‘DvsS’ and ‘SNP_Z’ maps, respectively.

Both maps were developed from the dataset obtained with the 9K SNP array and filtered according to the parental alleles. Regarding the DvsS map, only segregating SNPs heterozygous in the F1 parent (SD40) but homozygous in both Z and S and monomorphic were used for the mapping. The SNP dataset was combined with the previous mapping dataset used in Illa *et al.* (2009), and the resulting dataset was used for further analysis. The second map (SNP_Z) was developed based on the segregating SNPs heterozygous in Z but homozygous in both SD40 and S and monomorphic.

Both maps were constructed using JoinMap 4.1 software (Van Ooijen, 2011). BcpxFy population-type codes were applied for the DvsS map, with x=2 and y=0; this corresponds to an advanced backcross 2 population (BC2). For the SNP_Z map, CP population-type codes were applied. Deviations from the expected Mendelian ratio of 1:3 and 1:1 for DvsS and SNP_Z, respectively, were tested using the chi-square-goodness of fit test (*P*<0.05). Linkage groups were established using a minimum 3.0 logarithm of odds (LOD) and an initial maximum recombination frequency of 0.35. Map construction was performed using the multipoint maximum likelihood-based algorithm (Jansen *et al.*, 2001). Genetic distances were calculated using the Kosambi mapping function (Kosambi, 1944), and Mapchart 2.2 software (Vooripps, 2002) was used to generate map figures.

In addition, an integrated physical map was built for each of the eight linkage groups including all of the SNPs present in the genetic maps mentioned above as well as markers with known positions mapped by Illa *et al.* (2009). Markers were placed on the map according to their positions in the peach genome sequence v2.0 (http://www.rosaceae.org/species/prunus_persica/genome_v2.0.a1).

### QTL analysis

The dataset, rescaled as explained above, was used for QTL detection for the 19 traits studied at the six percentages of fruit development and for the two genetic maps using R software (R Development Core Team 2011, ‘qtl’ library). Among the 114 datasets studied (19 traits, six stages), 33 followed a normal distribution, 56 were transformed to follow a normal distribution and 25 others could not be transformed to follow a normal distribution. Concerning the latter datasets, a nonparametric method was used for the QTL detection based on the method described by Kruglyak and Lander (1995). Regarding the datasets that follow a normal distribution, QTL detection was performed by marker regression (Soller *et al.*, 1976). Considering the particular characteristics of the BC2 progeny, the analysis was performed only at the genetic markers (no interval mapping), and individuals with missing genotypes were discarded.

### Projection on the physical map and synthetic QTL compilation

BioMercator software v4.2 (Arcade *et al.*, 2004) was used to make links between each of the genetic maps and the SNP-based physical map mentioned above, and QTLs detected on DvsS and SNP_Z maps were projected together on the physical map. For a given trait, if QTLs were detected in the same region at the different fruit developmental dates, they were compiled into a single QTL (synthetic QTL), and the smallest common region between the QTL was kept to define the associated confidence interval.

### Identification of candidate genes

From the functional annotation of the *Prunus persica* genome v2.0 (https://www.rosaceae.org/species/prunus_persica/genome_v2.0.a1), a systematic search was conducted to compile the location on the physical map of all genes coding for enzymes or transporters directly linked to sugar metabolism. A co-location was declared between a QTL and a candidate gene when the latter was located in the confidence interval of the synthetic QTL. Map figures were generated using MapChart 2.2 software (Voorrips, 2002).

## Results

### Genotyping and genetic map

Out of the 8144 working SNPs from the 9K SNP peach array, 3194 (39.2%) did not fulfil the filtering criteria and 1350 (16.6%) were homozygous in the population, leaving 3600 segregating SNPs (44.2%) useful for mapping. These SNPs were separated into two classes: one composed of the SNPs heterozygous in ‘Zephyr’ and the other composed of the SNPs homozygous in ‘Zephyr’. They contained 1739 (21.4%) and 1861 (22.8%) SNPs, respectively (Supplementary Fig. S3). The SNPs included in these two classes were used separately to construct the DvsS and the SNP_Z maps after additional filtering steps (Supplementary Figs S4, S5).

The DvsS map was built from 123 individuals. Among the 962 SNPs selected (Supplementary Fig. S3), 741 were mapped to the eight linkage groups. Most of the mapped SNPs cosegregated in clusters and, as a consequence, were distributed among 209 independent loci. In addition, 139 markers (72 SSRs, 23 AFLPs, 35 RFLPs and one phenotypic character) from the previous mapping dataset (Illa *et al.*, 2009) were added to the mapping framework, resulting in 340 independent loci spanning a total length of 420 cM. This corresponded to an average interval of 1.24 cM between markers. Based on the positions of the markers mapped on the linkage groups, the calculated physical map length was 213 795 364bp in total, corresponding to 94.73% of the peach genome v2.0 (Supplementary Table S1). No genome rearrangement was observed, and marker positions on the linkage groups were consistent with their positions in the peach genome v2.0.

With respect to the SNP_Z map, a total of 593 SNPs (7.3%) mapped to the eight linkage groups. The clustering of markers was particularly high in G2, in which as many as 182 SNPs cosegregated. This high number of cosegregating heterozygous markers in quite a large physical region (134 markers between 4.3 Mbp and 9.7 Mbp on the peach genome v2.0) could be explained by the low recombination rate observed, which maintained the heterozygosity. These 593 SNPs were distributed among 117 independent loci harbouring 7.3 SNP/locus on average, but 4.6 SNP/locus if G2 was excluded. They spanned a total length of 389.7 cM corresponding to an average interval of 3.3 cM between markers. However, G5 was almost missing, and only the upper part of G2 was mapped. In addition, three large gaps of 31.3 cM (G1), 22.7 cM (G2) and 52.6 cM (G6) remained, as well as six other gaps larger than 10 cM (G1, G3, G4, G7 and G8). The calculated physical map length was 150 230 193bp in total, corresponding to 66.56% of the peach genome v2.0 (Supplementary Table S1). The percentages of coverage varied among the linkage groups from the lowest coverage of 2.77% for G2 to the highest coverage of 91.37% for G6. SNP positions on the linkage groups conformed to their physical positions.

### Phenotyping data

The 106 individuals of the BC2 progeny were phenotyped for 19 traits, including fruit fresh weight, sugar concentrations, acid concentrations and enzyme capacities related to sugar metabolism, at six stages during fruit development. The population displayed large variations both in terms of levels of contents and capacities and in terms of temporal evolutions (Supplementary Fig. S6). Sucrose concentration rapidly increased during fruit growth and became the main sugar. Glucose concentration was maintained at a fairly constant level, and fructose concentration declined before rising again. Sorbitol, the least abundant of the measured sugars, increased slowly during growth and decreased slightly at maturity. The enzymatic capacities displayed a large range of levels depending on the enzyme. In contrast to temporal variations of metabolites, those observed for the enzymatic capacities were rather reduced.

### QTL location

**Fig. 1.**
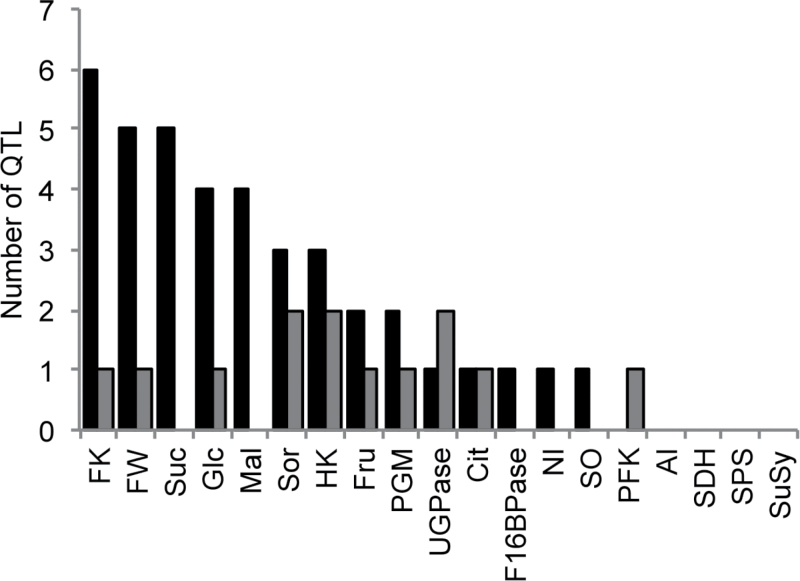
The number of synthetic QTL detected for all 19 traits. Abbreviations: AI, acid invertase; Cit, citrate; F16BPase, fructose-1,6-bisphosphatase; FK, fructokinase; Fru, fructose; FW, fresh weight; Glc, glucose; HK, hexokinase; Mal, malate; NI, neutral invertase; PFK, ATP-phosphofructokinase; PGM, phosphoglucomutase; SDH, sorbitol dehydrogenase; SO, sorbitol oxidase; SPS, sucrose phosphate synthase; Sor, sorbitol; Suc, sucrose; SuSy, sucrose synthase; UGPase, UDP-glucose pyrophosphorylase. The QTLs in black were detected from the DvsS polymorphism and the QTLs in grey from the Z polymorphism.

The maximum number of distinct QTLs detected for a single trait was seven, which were observed for FK, while six QTLs were observed for FW and five QTLs were observed for sucrose (Fig. 1). Sucrose is the main sugar in peach and exhibited great phenotypic variability in the BC2 progeny. Interestingly, a large number of QTLs for enzymes (notably, FK, HK, PGM and UGPase) were detected, which suggested complex genetic determinism of enzymatic capacities. However, the percentage of phenotypic variation explained by a single QTL was lower for enzymatic capacities than for metabolite concentrations (Table 2). In addition, no QTL was detected for four enzymes (AI, SDH, SPS and SuSy) (Fig. 1).

**Table 2.** QTL descriptions

**Trait**	**LG**	**Stages**	**% of trait variability explained by the QTL**	**Wild allele effect (D**)	**Map**	**Candidate genes co-located with the QTL**
**Glc**	1	12	[3.35–5.01]	+	DvsS	**Sugar transporter** (Prupe.6G151200, Prupe.1G133300, Prupe.2G118600, Prupe.3G066300); **Invertase** (Prupe.1G111800); **Invertase inhibitor** (Prupe.1G105400, Prupe.1G113800, Prupe.1G114500, Prupe.1G118800, Prupe.1G123500, Prupe.1G123700, Prupe.1G123800, Prupe.1G131900, Prupe.1G132000, Prupe.1G132300)
**Suc**	1	56	[14.23–19.11]	+	DvsS	**Sugar transporter** (Prupe.1G133300); **Invertase** (Prupe.1G111800); **Invertase inhibitor** (Prupe.1G105400, Prupe.1G113800, Prupe.1G114500, Prupe.1G118800, Prupe.1G123500, Prupe.1G123700, Prupe.1G123800, Prupe.1G131900, Prupe.1G132000, Prupe.1G132300); **SuSy** (Prupe.1G131700)
**Sor**	1	1	16.64	-	DvsS	**Sugar transporter** (Prupe.1G133300)
**UGPase**	1	1	14.33	-	DvsS	
**Fru**	1	123456	[47.76–86.24]	-	DvsS	**Sugar transporter** (Prupe.1G133300); **Invertase inhibitor** (Prupe.1G131900, Prupe.1G132000, Prupe.1G132300); **SuSy** (Prupe.1G131700)
**Mal**	1	12	[2.96–12.66]	+	DvsS	
**FW**	1	56	[5.41–6.15]	-	DvsS	
**UGPase**	1	45	[8.97–11.25]		SNP_Z	
**HK**	1	345	[11.13–14.02]		SNP_Z	**HK** (Prupe.1G366000)
**HK**	1	4	7.51	-	DvsS	
**PGM**	1	2	10.96	-	DvsS	
**FK**	1	1	4.91	-	DvsS	
**Cit**	2	345	[7.48–17.78]	-	DvsS	
**Mal**	2	345	[12.50 -15.16]	+	DvsS	
**Sor**	2	6	12.57		SNP_Z	**Sugar transporter** (Prupe.2G325500, Prupe.2G245600, Prupe.8G224400, Prupe.5G006100)
**F16BPase**	3	45	[8.12–9.25]	+	DvsS	
**FK**	3	5	4.1	+	DvsS	
**UGPase**	3	6	14.33		SNP_Z	**UGPase** (Prupe.3G015000)
**FK**	4	56	[11.23–12.99]		SNP_Z	
**Fru**	4	12345	[0.3–1.64]	-	DvsS	**Invertase inhibitor** (Prupe.4G001200, Prupe.4G025200); **Sugar transporter** (Prupe.7G223700, Prupe.5G128400, Prupe.5G125100, Prupe.3G152800, Prupe.4G072300, Prupe.8G020100);
**Fru**	4	12	13.5		SNP_Z	**Sugar transporter** (Prupe.4G083700, Prupe.4G008300, Prupe.7G185800, Prupe.7G247800); **Invertase** (Prupe.4G206400); **SDH** (Prupe.4G240300)
**FW**	4	2	13.16		SNP_Z	
**Glc**	4	234	[0.03–0.41]	-	DvsS	**Sugar transporter** (Prupe.6G331800, Prupe.4G127200)
**Glc**	4	56	[11.51–15.88]		SNP_Z	**Sugar transporter** (Prupe.4G083700, Prupe.4G008300, Prupe.7G185800); **Invertase** (Prupe.4G206400)
**HK**	4	6	13.5		SNP_Z	**HK** (Prupe.4G256200)
**Sor**	4	3456	[9.37–22.98]		SNP_Z	**Sugar transporter** (Prupe.5G125100, Prupe.3G152800, Prupe.4G072300, Prupe.8G020100, Prupe.6G331800, Prupe.4G127200, Prupe.1G516200, Prupe.4G083700, Prupe.4G008300, Prupe.7G185800, Prupe.7G247800); **SDH** (Prupe.4G240300)
**Cit**	5	6	[13.53–39.76]		SNP_Z	
**FK**	5	12	[2.59–6.08]	+	DvsS	
**FK**	5	35	[9–10.08]	+	DvsS	
**Glc**	5	1235	[2.47–13.8]	-	DvsS	**Sugar transporter** (Prupe.5G175500); **Invertase inhibitor** (Prupe.5G189700)
**HK**	5	34	[7.27–7.43]	-	DvsS	
**Sor**	5	2	11.01	+	DvsS	**Sugar transporter** (Prupe.1G156700, Prupe.6G183300, Prupe.5G083900, Prupe.7G185700, Prupe.7G234100, Prupe.2G306600, Prupe.4G197800, Prupe.4G155700, Prupe.5G091100, Prupe.6G187000, Prupe.4G127800, Prupe.5G175500);
**Suc**	5	1	2.34	+	DvsS	**Invertase** (Prupe.5G075600); **Sugar transporter** (Prupe.7G185700, Prupe.7G234100, Prupe.2G306600, Prupe.4G197800, Prupe.4G155700, Prupe.5G091100, Prupe.6G187000, Prupe.4G127800, Prupe.5G175500); **Invertase inhibitor** (Prupe.5G048600, Prupe.5G048900, Prupe.5G076800, Prupe.5G076900, Prupe.5G112600, Prupe.5G189700)
**FK**	6	56	[8.76–16.36]	-	DvsS	
**FW**	6	123	[11.88–19.17]	+	DvsS	
**HK**	6	56	[13.93–16.23]	+	DvsS	**HK** (Prupe.6G212100)
**PGM**	6	56	[10.37–10.66]	+	DvsS	
**Suc**	6	23	[3.4–7.54]	-	DvsS	**Sugar transporter** (Prupe.8G181000, Prupe.6G211400); **Invertase inhibitor** (Prupe.6G197400)
**FW**	7	456	[5.3–15.63]	-	DvsS	
**Glc**	7	1234	[17.41 52.17]	-	DvsS	**Sugar transporter** (Prupe.8G077700, Prupe.1G220700, Prupe.6G070700); **Invertase inhibitor** (Prupe.7G190300, Prupe.7G190400, Prupe.7G190500, Prupe.7G190700, Prupe.7G192400, Prupe.7G193700); **SuSy** (Prupe.7G192300)
**Mal**	7	2345	[11.54–26.99]	+	DvsS	
**NI**	7	3	8.29	+	DvsS	
**PGM**	7	1	13.35		SNP_Z	
**SO**	7	6	11.36	-	DvsS	
**Sor**	7	2	7.84	-	DvsS	**Sugar transporter** (Prupe.8G077700, Prupe.1G220700, Prupe.6G070700, Prupe.8G017500, Prupe.7G231500, Prupe.1G584600, Prupe.5G027100, Prupe.1G144800);
**Suc**	7	12356	[9.11–54.7]	+++--	DvsS	**Sugar transporter** (Prupe.8G077700, Prupe.1G220700, Prupe.6G070700); **Invertase inhibitor** (Prupe.7G190300, Prupe.7G190400, Prupe.7G190500, Prupe.7G190700, Prupe.7G192400, Prupe.7G193700); **SuSy** (Prupe.7G192300)
**Mal**	8	34	[2.69–5.91]	+	DvsS	
**PFK**	8	345	[12–13.38]		SNP_Z	
**Suc**	8	56	[4.6–6.64]	+	DvsS	**Sugar transporter** (Prupe.8G076100, Prupe.4G042700, Prupe.8G052700, Prupe.1G156900); **Invertase inhibitor** (Prupe.8G038100)

**Fig. 2.**
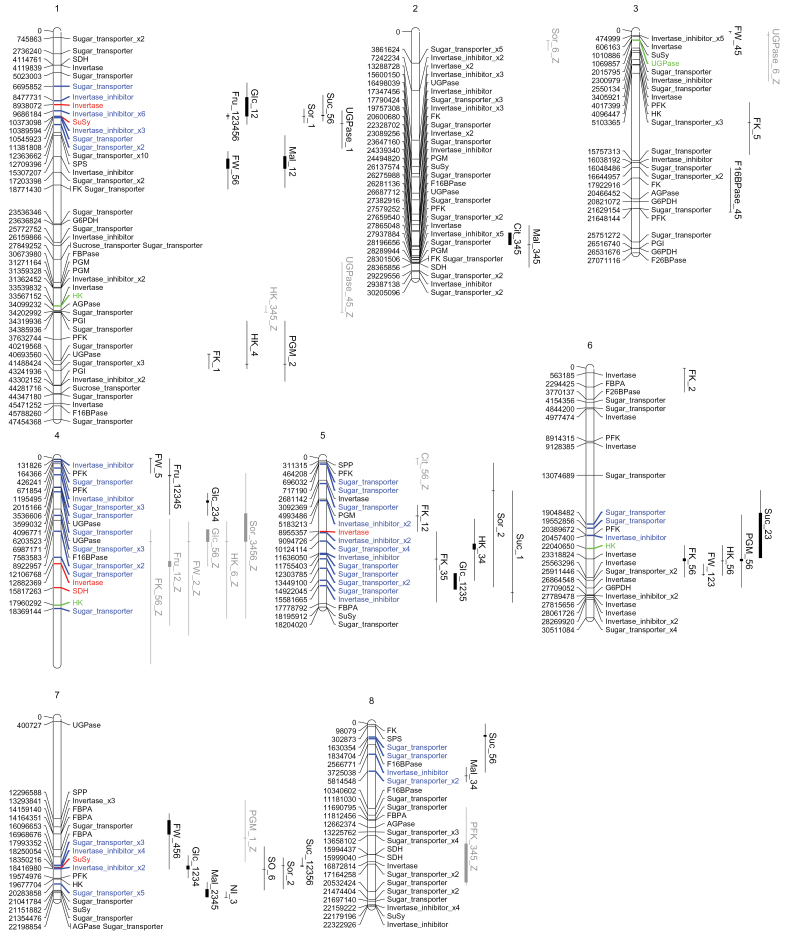
Location on the physical map of the synthetic QTL related to the 19 traits studied. Abbreviations: AI, acid invertase; Cit, citrate; F16BPase, fructose-1,6-bisphosphatase; FK, fructokinase; Fru, fructose; FW, fresh weight; Glc, glucose; HK, hexokinase; Mal, malate; NI, neutral invertase; PFK, ATP-phosphofructokinase; PGM, phosphoglucomutase; SDH, sorbitol dehydrogenase; SO, sorbitol oxidase; SPS, sucrose phosphate synthase; Sor, sorbitol; Suc, sucrose; SuSy, sucrose synthase; UGPase, UDP-glucose pyrophosphorylase. The numbers following the trait names refer to the stages at which the QTL was detected (1, 40% of development; 2, 52%; 3, 64%; 4, 76%; 5, 88%; 6, 100%). Z at the end of name of the QTL indicates that the QTL was detected on the ‘SNP_Z’ map. Only candidate genes and their locations (pb) are represented in the eight linkage groups; SNP markers have been discarded. In green, candidate genes coding for enzymes that co-locate with QTLs for capacity of the same enzymes; in red, candidate genes coding for enzymes that co-locate with QTLs for sugars that are substrates or products in the enzymatic reactions catalysed by these enzymes. In blue, candidate genes coding for sugar transporters or enzyme inhibitors that can have an impact on sugar metabolism and co-locate with sugar QTLs.

The 52 QTLs detected in this analysis were distributed over the entire genome (see Fig. 2 and Table 2 for an overview). Regarding the QTLs linked to the Z polymorphism, linkage group 4 harboured half of those detected on the SNP_Z map. In addition to this hotspot, several clusters of QTLs emerged from this analysis. On linkage groups 1 and 7, the clusters included QTLs for the four sugars together and for three sugars, respectively, with other traits.

This study provides an almost complete overview of sugar metabolism during peach development. The percentage of trait variability explained by all QTLs detected is between 7.3% and 87.5%, depending on the traits and stages (Supplementary Fig. S7).

### QTL stability during fruit development

**Fig. 3.**
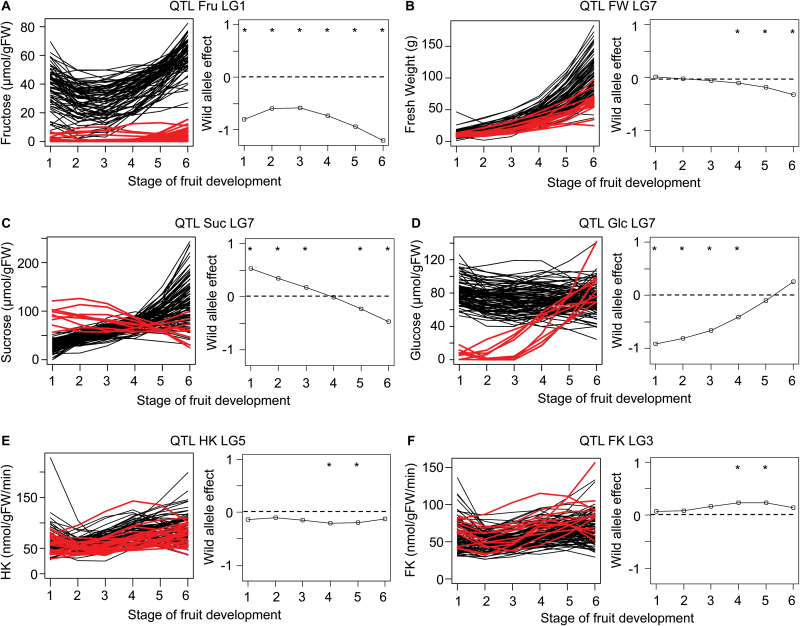
Evolution of the wild allele effect during fruit development. For the six QTLs represented (A–F), the QTL name and the LG are specified, and two panels are plotted. On the left, a fit of the six stages of fruit development is plotted for all genotypes; genotypes with the wild allele are in red. In the right panel, the wild allele effect is represented as a proportion of trait variation. Stars specify the stage at which the QTL is significant. Abbreviations: FK, fructokinase; Fru, fructose; FW, fresh weight; Glc, glucose; HK, hexokinase; Suc, sucrose.

Allele effects changed during fruit development and in different ways according to the QTL. Some examples are shown in Fig. 3. The QTL represented in Fig. 3A displayed a strong negative effect on fructose concentration throughout fruit development. This QTL regulates the fructose-to-glucose ratio and corresponds to the QTL FRU published by Quilot *et al* (2004). In contrast to this stability, the D allele at the QTL for FW shown in Fig. 3B had no effect in the first stages but then displayed a negative effect that increased with fruit development. Some QTLs had an important effect on the time-course shape of traits studied as shown in Fig. 3C and Fig. 3D, with a shift from positive to negative effects or vice versa, of the allele D effect during development.

**Fig. 4.**
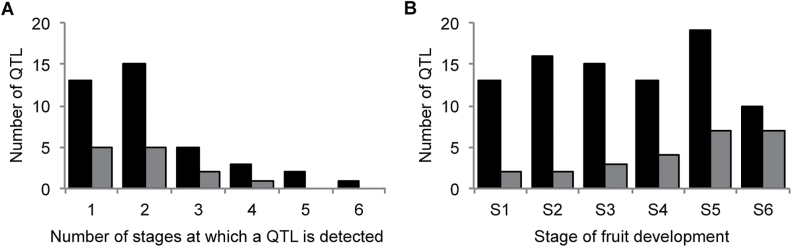
Number of QTLs detected (A) simultaneously at different stages of fruit development, and (B) at each of the rescaled values. The QTLs in black were detected from the DvsS polymorphism and the QTLs in grey were detected from the Z polymorphism.

Most of the QTLs were not detected at some stages in fruit development because their effect was not constant. Only one QTL was detected at all six developmental stages (Fig. 4A), and it corresponded to the above mentioned FRU QTL on linkage group 1 (Fig. 3A). Otherwise, in most cases (33% for the DvsS polymorphism and 38% for the Z polymorphism), QTLs were detected only for a single stage (Fig. 4A). The number of QTLs detected for each stage was between 2 and 19, with a higher number for stages 2 and 5 for the DvsS polymorphism and for stages 5 and 6 for the Z polymorphism (Fig. 4B). At each stage, there are different QTLs that are detected only at that specific stage. Panels E and F of Fig. 3 illustrate QTLs with negative and positive effects, respectively, at only a few stages of development.

### Functional co-location between QTLs and between QTLs and candidate genes

The identification of functional genes based on their automated annotation (The International Peach Genome Initiative, 2013) and the comparison of their locations with QTLs allowed for the identification of candidate genes that could be responsible for variations in sugar metabolism.

Four QTLs linked to sugar concentration co-located with QTLs for an enzyme catalysing the synthesis or degradation of the specific sugar. On linkage group 4, HK and Glc QTLs were identified from the Z polymorphism at stages 5 and 6 as well as Fru and FK QTLs. On linkage groups 5 and 7, co-locations were identified from the DvsS polymorphism between Sor and SO QTLs and between Glc and HK QTLs, respectively. In each case, the co-locations occurred between a sugar and an enzyme catalysing its hydrolysis, offering functional hypotheses to explain the phenotypic variability observed.

To delve deeper into the functional analysis of the QTLs, 296 candidate genes were identified from their functional annotation as enzymes related to sugar metabolism (99), sugar transport (133) or invertase inhibitors (64) (Supplementary Table S2). Four QTLs controlling enzyme capacities co-located with a gene coding for the same enzyme (Table 2, Fig. 2 in green), which made them good candidate genes for both positional and functional aspects. In addition to this, there were six QTLs for sugars that co-located directly with genes of enzymes involved in their synthesis or degradation (Table 2, Fig. 2 in red).

## Discussion

### Marker density and population size – factors controlling QTL detection

The complex structure of the studied population limited the number of SNPs that were useful for the construction of the genetic maps. However, the DvsS map used in this study is fairly accurate compared to the maps usually used for genetic mapping studies in *Prunus* (Salazar *et al.*, 2014). Therefore, the limiting factor impairing QTL detection and resolution in this study might be the population size. However, in *Prunus* species and stone fruit in general, the number of hybrids produced by trees is limited by the single seed of a fruit. In addition, the length of the juvenile period and the necessity of maintaining an orchard for several years results in significant orchard management costs. In practice, these constraints result in rather small population sizes typically used for genetic mapping studies in *Prunus* as reviewed by Salazar *et al.* (2014). The range extends from 48 to 270 descendants in peach population with a median of 77. Consequently, we believe the study presented here including 106 genotypes is reasonable. In addition, the BC2 population studied displays a huge range of variations of the traits analysed, as reviewed for sugar content in Cirilli *et al.* (2016), which makes it of particular interest even with a limited number of individuals.

### A difficult compromise to establish the experimental design

In addition to the population size, discussed above, year or site replications are considered essential to verify the reproducibility of the results. Similarly, replicated phenotypic measurements are recommended to ensure accurate evaluation. This is easier to achieve for an annual crop (e.g. tomato or melon) than for a perennial tree species. Consequently, we faced a difficult compromise to establish the experimental design. In *Prunus* species, replication is generally not affordable in the same orchard, due to orchard management costs and the maintenance of the trees over multiple years. In general, replication studies are achieved by multi-year phenotyping, which is much more frequent in the peach QTL literature (Salazar *et al.*, 2014). In our case, the phenotyping was a major constraint in terms of both time and cost. It was not possible to repeat the analysis a second year or to increase the number of samples analysed per individual and stage. Knowingly, we chose to maximize the number of individuals, the number of development stages and the number of traits studied to the detriment of the biological replicates and the year repetitions. This design was meant to explore the entirety of sugar metabolism throughout fruit development to detect dynamic QTLs and to address this missing aspect of the literature. As the population had already been the subject of a published study for quality traits (Quilot *et al.*, 2004), it is possible to compare the results at maturity from the current and previous studies, and assess yearly stability (see below).

### Identification of loci governing fruit sugar metabolism

Several QTLs were detected for all of the traits studied, namely sugar concentrations, acid concentrations, FW, and enzyme capacities for the different stages of fruit development. The distribution of the QTLs detected revealed that sugar concentrations and enzyme capacities are under the control of several loci distributed among the eight linkage groups. This is in accordance with results from previous studies that aimed to detect sugar QTLs on peach fruit at maturity only. Fru QTLs were found in all linkage groups; Glc QTLs were found in linkage groups 1, 2, 4, 5, 7 and 8; Sor QTLs were found in linkage groups 1, 2, 4, 5 and 6; and Suc QTLs were found in linkage groups 3, 5, 6 and 7 (Sosinski *et al.*, 1998; Dirlewanger *et al.*, 1999; Etienne *et al.*, 2002; Quilot *et al.*, 2004). Our data complete this picture with additional QTLs that have never been reported before (Supplementary Fig. S8). Our data reveal the presence of Sor QTLs on linkage groups 1 and 7, a Glc QTL on linkage group 1 and a Suc QTL on linkage group 1 that is detectable at maturity. All of these studies, except the study by Sosinski *et al.* (1998), found a Fru QTL on linkage group 4. In our study, a Fru QTL was detected based on the DvsS polymorphism. Due to the presence of QTLs with high effects on linkage group 1 (47.76 to 86.24%), the QTL on linkage group 4 only explained 0.3 to 1.64% of the fructose variability (depending on the stage). Another Fru QTL on linkage group 4 was also detected based on the Z polymorphism and explained 13.5% of fructose phenotypic variation.

In addition, we detected QTLs on the SNP_Z map, despite the low polymorphism of this variety. As observed by Quilot *et al.* (2004), linkage group 4 displays many QTLs at maturity coming from Z, which includes Glc QTL and Sor QTL. A Sor QTL and a Cit QTL were also detected at maturity on linkage groups 2 and 5, respectively. These data uncovered interesting alleles from Z for breeding purposes. Indeed, considering the high level of sugars of Z fruits, one might have expected that the best alleles were fixed in the Z genome. Therefore, genetic progress may still be possible in the case of additive effects by doubling the best alleles at the QTLs.

### Phenotyping accuracy guarantees QTL mapping consistency

Even though a large number of QTLs were detected, this study highlights three shortcomings that deserve special attention when performing a QTL analysis. They all concern different aspects of phenotyping accuracy or, more precisely, stability evaluation across years, precision of evaluation and timing of sampling.

Despite the large number of QTLs detected, several QTLs detected by Quilot *et al.* (2004) at maturity were not detected in the present study although the same progeny were studied. However, these QTL (except Mal) were detected in only one year out of the two years studied by Quilot *et al.* (2004) suggesting a low stability of these QTL presumably due to differences in other factors between years. In addition, because the map used in this previous study was not dense, QTL accuracy should be treated with caution. Nevertheless, 11 QTLs for FW, sugar content and acid content were detected in both analyses (Quilot *et al.*, 2004 and the present study), which confirms their stability. Moreover, in the present study, among the 16 new QTLs found compared to Quilot *et al.* (2004), four were detected at maturity, which may result either from the low stability of these QTLs between years or from the improvement of the genetic map.

This question of stability often arises with complex traits resulting from different processes affected by environmental factors. This resulted in a relatively low number of QTLs. One way to take into account this variability is to further investigate the processes and decipher the component traits that mainly cause the variability. Such dissection of complex traits into elementary physiological processes may be achieved through process-based modelling. For instance, process-based models have been successfully used to study the co-locations of QTLs for model parameters associated with specific processes and for peach fruit traits (Quilot *et al.*, 2005).

In addition to the stability of the QTLs, a second difficulty concerns the accuracy and precision of the phenotyping data. Remarkably, in our study, more QTLs were detected for traits that displayed high homogeneity between biological and technical repetitions (i.e. Suc, Glc, Fru, FK and HK). For some enzymes and citrate concentrations, the variability observed between technical replicates may explain the low number of QTLs detected. This indicates the importance of technical replicates and the need for developing high quality robot-based phenotyping platforms (Gibon *et al.*, 2004). The last major issue brought up by this study has to do with sampling, namely with the difficulty of identifying identical physiological stages during fruit development for stone fruit in general and when large genetic variability is studied in particular. In general, fruit growth duration is usually simply divided into equal periods. However, in the population studied, the duration of fruit development was highly variable depending on the genotype, and the prediction of maturity date was somewhat imprecise, resulting in marked time lags between successive sampling dates. To tackle this difficulty, a rescaled dataset was calculated from the observed dataset on the basis of percent of fruit development duration (see ‘Materials and methods’ section).

### Identification of functional and positional candidate genes

Genes coding for enzymes were found to co-localize with QTLs for activity of the same enzymes and became interesting candidate genes to explain the variation of these enzyme activities (Causse *et al.*, 1994; Thévenot *et al.*, 2005). Maximal enzyme activities depend on the number of molecules of the enzymes synthesized, which is linked to the transcript levels of their encoding structural genes. The level of transcription of a gene is regulated by gene expression that involves both cis-regulatory elements near the gene and trans-acting factors. In the case of cis-regulation, co-location between the structural gene and the QTL for enzyme capacity may be observed (Keurentjes *et al.*, 2008).

In contrast, variations in trans-regulation result in co-location of QTLs for enzyme capacity with genes coding for regulatory proteins. However, such genes are still poorly studied at the genetic level; therefore, few genes are annotated as such on the peach genome, except some enzyme inhibitors, especially invertase inhibitors.

Another scenario observed is the co-location between a structural gene and a QTL controlling the related sugar but no QTL for activity of the corresponding enzyme. This particular situation may be explained by the role of the level of affinity of an enzyme in the production of the metabolite. Enzyme affinity for a substrate, given by the Michaelis constant (*K*_*M*_), is linked to the conformation of the protein that results from the gene sequence coding for the enzyme, but it can also depend on allosteric regulation. For instance, allosteric inhibitors induce a conformational modification that changes the shape of the active site and reduces the affinity of the enzyme for its substrate. Long and fastidious experiments are required to measure *K*_*M*_ and phenotyping *K*_*M*_ in a progeny is unrealistic. In our study, several co-locations between a structural gene and a QTL for the related sugar were found. For example, one outstanding co-location occurs on linkage group 1 between a Suc QTL (with a positive effect of the wild allele) and a Fru QTL (with a negative effect of the wild allele) and a gene coding for SuSy. That may correspond to cases of DNA polymorphisms of the structural gene of SuSy that no longer permit the synthesis of the functional enzyme that cleaves sucrose into fructose.

In the case of allosteric regulation, the sugar QTLs may co-locate with genes coding for regulators or other enzymes controlling (de)phosphorylation. Unfortunately, most of these genes may have unknown functions in the peach genome annotation. These cases may correspond to the QTL for sugar for which no candidate genes were recorded.

Finally, most of the annotated genes involved in sugar metabolism did not co-localize with a QTL. This can be explained by a lack of polymorphism in the progeny or a lack of power for QTL detection (insufficient precision of measurements discussed below or number of individuals studied). However, this can also result from the fact that variations in enzyme activities are generally slight compared to variations in metabolites. Biais *et al.* (2014) and Desnoues *et al.* (2014) recently highlighted the low variations of enzymatic activities under contrasting environments and genetic variability.

### Changing effects of alleles during fruit development revealed by dynamic QTLs

This study marks the first time that dynamic QTLs for sugar metabolism have been detected in peach. Previous studies have looked for dynamic QTLs during seed or fruit development in other species (Thévenot *et al.*, 2005; Costa *et al.*, 2010; Han *et al.*, 2011; Sun *et al.*, 2012; Jiang *et al.*, 2013). They all revealed that different QTLs were detected depending on time and indicated that differential gene expression may be involved. In the same way, in the present study only one QTL was present at the six developmental stages studied. This QTL present on linkage group 1 corresponds to the locus FRU detected by Quilot *et al.* (2004) at maturity. With this locus, only seven of 52 QTL were detected at four stages or more. Remarkably, these QTLs were not randomly distributed along the genome: three of them were located on the cluster on linkage group 7. This highlights the importance of this genomic region for sugar metabolism throughout fruit development.

The variability in QTL detection across developmental stages arises primarily from the fact that the effect of the D allele is not stable during fruit development. Indeed, for some QTLs, it changes drastically. For example, the Suc QTL on linkage group 7 exhibits a switch of the D allele effect from significantly positive (stages 1, 2 and 3) to significantly negative (stages 5 and 6) (Fig. 3C). Interestingly, this QTL co-localizes with a Glc QTL that exhibits an inverse trend of the D allele effect (Fig. 3D), but no Glc QTL was detected at maturity. These two QTLs may be the result of the same gene having an opposite effect on Suc and Glc and may present a switch of its expression in the middle of fruit growth. The genes encoding SuSy that catalyse sucrose cleavage into fructose and UDP-glucose present in the region of this QTL would be good functional candidate genes. Further study is needed to confirm this assumption and to identify the functional gene underlying these QTLs.

### *Prunus davidiana* alleles display changing effects during growth that might impair their use in breeding

From the perspective of breeding, D was used as a source of resistance to pests and diseases but has very poor agronomical attributes (Moing *et al.*, 2003). Interestingly, Quilot *et al.* (2004) reported positive effects of the D allele at QTLs on linkage groups 4, 5 and 7 on glucose concentration at maturity. Here, we reported negative effects at the same loci on linkage group 7 during fruit development, but no Glc QTL was detected at maturity. These loci display negative effects at early stages that become non-significant and sometimes reverse towards positive values during growth, without reaching significant levels at maturity. An extra harvest of the fruits a few days later may have resulted in the discovery of the positive effect. This is not without impact for breeding perspectives. Indeed, the positive desired effect of an allele may become negative depending on climatic conditions or harvest date. Moreover, the Glc QTL on linkage group 7 co-localizes with a Suc QTL for which the D allele has a negative effect at maturity. Therefore, given this particularity and the low sweetness of Glc (0.75 compared to sucrose; Pangborn, 1963), the Glc QTL on linkage group 7 detected by Quilot *et al.* (2004) may not be relevant for peach fruit quality improvement.

This particular case opens the door to a series of questions of interest for breeding. Indeed, the particular feature relating to reverse effects during growth, uncovered in this study, is not specific to the wild species *P. davidiana* because a similar pattern was found with the Z polymorphism for Glc QTL on linkage group 4. To the best of our knowledge, no other examples are available in the literature, and further studies are needed to identify the mechanisms responsible for these reversions. In this way, it may be envisaged to move forwards the reversion to get a stable effect within a time slot around harvest at maturity. The study of gene expression during growth would most likely aid in deciphering the mechanisms controlling these features. In the same way, this discovery raises questions regarding the QTL effect after harvest and the common strategy of basing selection upon effects detected at maturity only. In future breeding strategies, it will certainly be of great interest to include the monitoring of the QTL effect during fruit development and post-harvest.

## Conclusion

The present study provides a broad overview of the genetic control of sugar metabolism during peach fruit development. QTLs were detected for FW, metabolites and maximal enzyme activities at each developmental stage studied. Several co-locations were found between candidate genes, QTLs for enzyme activities and/or QTLs for metabolites that lead to functional hypotheses. However, more studies are needed to validate the candidate genes underlying these QTL and to uncover their functions. Finally, we demonstrated the instability of the QTL effect during fruit development, which can have major implications for fruit breeding.

## Supplementary data

Supplementary data are available at *JXB* online.

Table S1. Characteristics of the genetic linkage maps derived from the BC2 progeny.

Table S2. Description of the genes shown in Fig. 2.

Fig. S1. Peach sugar metabolism.

Fig. S2. Example of rescaling data: sucrose concentration (μmol.gFW-1) during fruit development (in%) for two genotypes.

Fig. S3. Flow chart of the filtering procedure performed to select SNPs that were useful for the construction of the DvsS and the SNP_Z maps.

Fig. S4. Location on the genetic DvsS map (cM) of the QTLs related to the 19 traits studied.

Fig. S5. Location on the genetic SNP_Z map (cM) of the QTLs related to the 19 traits studied.

Fig. S6. Boxplot of (A) sugar and acid concentrations (µmol.gFW^−1^), (B) enzymatic capacities (µmol.gFW^−1^min^−1^) and (C) fresh weight (FW) (g) at the six stages of fruit development for all genotypes studied.

Fig. S7. Distribution of the total percentage of trait phenotypic variation explained by all of the QTLs detected for a trait.

Fig. S8. Schematic representation of QTLs for sugar, acid and fresh weight from the literature reported together with QTLs from the present study.

Supplementary Data
