# Supplementary material for: Dynamic QTLs for sugars and enzyme activities provide an overview of genetic control of sugar metabolism during peach fruit development
Source: J Exp Bot. 2016 Apr 25;67(11):3419–31. doi: 10.1093/jxb/erw169 (PMC4892732; doi:10.1093/jxb/erw169)
Supplement: Supplementary Data [file supp_67_11_3419__index.html]

Dynamic QTLs for sugars and enzyme activities provide an overview of genetic control of sugar metabolism during peach fruit development — Dynamic QTLs for sugars and enzyme activities provide an overview of genetic control of sugar metabolism during peach fruit development — Supplementary Data 

# Dynamic QTLs for sugars and enzyme activities provide an overview of genetic control of sugar metabolism during peach fruit development

## Supplementary Data

Data files

- supplementary\_tables\_S1\_S2\_supplementary\_figures\_S1\_S8.pdf - Supplementary Data
