# Supplementary material for: Dynamic QTLs for sugars and enzyme activities provide an overview of genetic control of sugar metabolism during peach fruit development
Source: J Exp Bot. 2016 Apr 25;67(11):3419–31. doi: 10.1093/jxb/erw169 (PMC4892732; doi:10.1093/jxb/erw169)
Supplement: Supplementary Data [file supp_erw169_supplementary_tables_S1_S2_supplementary_figures_S1_S8.pdf]

Dynamic QTLs for sugars and enzyme activities provide an overview of genetic control of sugar metabolism during peach fruit development
Elsa Desnoues, Valentina Baldazzi, Michel Génard, Jehan-Baptiste Mauroux, Patrick Lambert, Carole Confolent, Bénédicte Quilot-Turion

SUPPLEMENTARY MATERIAL

Supplementary Table S1: Characteristics of the genetic linkage maps derived from the BC2 progeny

Genetic map	Linkage group	Total # loci[a]	# SNP loci[a]	Total # SNPs	# SNP /locus[a]	# other markers loci[a]	Total # other markers	Map size (cM)	Marker interval (cM)	Physical size (bp)[b]	Coverage (%)[b]
'DvsS'											
	1	67	34	126	3.7	33	39	86.5	1.29	46,815,236	97.84
	2	38	30	122	4.1	8	8	42.7	1.12	29,975,404	98.58
	3	34	19	64	3.4	15	15	55.8	1.64	25,756,096	94.11
	4	46	26	129	5	20	22	56.2	1.22	23,489,337	90.89
	5	45	28	76	2.7	17	17	49.8	1.11	17,909,179	96.82
	6	42	27	82	3	15	15	50.9	1.21	28,805,581	93.62
	7	38	21	57	2.7	17	17	41.2	1.08	20,628,629	92.14
	8	30	24	85	3.5	6	6	36.9	1.23	20,415,896	90.44
	Total	***340***	***209***	***741***	***3.5***	***131***	***139***	***420***	***1.24***	***213,795,364***	***94.73***
'SNP_Z'											
	1	*7*	*7*	*24*	*3.4*	*NA*	*NA*	*53.7*	*7.67*	*26,754,694*	*55.91*
	2	*7*	*7*	*182*	*26*	*NA*	*NA*	*9.2*	*1.31*	*8,410,254*	*2.77*
	3	*18*	*18*	*60*	*3.3*	*NA*	*NA*	*52*	*2.89*	*23,061,269*	*84.26*
	4	*32*	*32*	*90*	*2.8*	*NA*	*NA*	*79*	*2.47*	*22,788,046*	*88.18*
	5	*3*	*3*	*27*	*9*	*NA*	*NA*	*1.9*	*0.63*	*752,765*	*4.1*
	6	*12*	*12*	*57*	*4.7*	*NA*	*NA*	*68.1*	*5.67*	*28,111,784*	*91.37*
	7	*26*	*26*	*82*	*3.2*	*NA*	*NA*	*65.6*	*2.52*	*19,566,416*	*87.39*
	8	*12*	*12*	*71*	*5.9*	*NA*	*NA*	*60.2*	*5.02*	*20,784,965*	*92.07*
	Total	***117***	***117***	***593***	***7.3***	***NA***	***NA***	***389.7***	***3.33***	***150,230,193***	***66.56***

[a]Locus: a single position on the genetic linkage map in which several markers may co-segregate

[b]Physical size and coverage: as compared to peach genome v2.0 pseudo-molecules (http://www.rosaceae.org/species/prunus_persica/genome_v2.0.a1)

Supplementary Table S2: Description of the genes shown on Figure 2.

Name	Ch	Position min	Position max	Annotation
Prupe.4G127100	Pp01	735953	738511	Sugar transporter
Prupe.5G006600	Pp01	743305	745863	Sugar transporter
Prupe.1G156800	Pp01	2732518	2736240	Sugar transporter
Prupe.1G057900	Pp01	4111842	4114761	SDH
Prupe.1G058000	Pp01	4118992	4119839	Invertase
Prupe.8G224500	Pp01	5020692	5023003	Sugar transporter
Prupe.6G151200	Pp01	6690069	6695852	Sugar transporter
Prupe.1G105400	Pp01	8475392	8477731	Invertase inhibitor
Prupe.1G111800	Pp01	8933938	8938072	Invertase
Prupe.1G113800	Pp01	9041293	9042145	Invertase inhibitor
Prupe.1G114500	Pp01	9074127	9074847	Invertase inhibitor
Prupe.1G118800	Pp01	9314174	9315654	Invertase inhibitor
Prupe.1G123500	Pp01	9684058	9686184	Invertase inhibitor
Prupe.1G123700	Pp01	9741098	9743331	Invertase inhibitor
Prupe.1G123800	Pp01	9751037	9753234	Invertase inhibitor
Prupe.1G131700	Pp01	10355300	10373098	SuSy
Prupe.1G131900	Pp01	10381907	10384156	Invertase inhibitor
Prupe.1G132000	Pp01	10387303	10389594	Invertase inhibitor
Prupe.1G132300	Pp01	10419659	10421796	Invertase inhibitor
Prupe.2G118600	Pp01	11370367	11374044	Sugar transporter
Prupe.3G066300	Pp01	11381808	11385053	Sugar transporter
Prupe.4G127400	Pp01	12052878	12057897	Sugar transporter
Prupe.8G248500	Pp01	12234605	12235403	Sugar transporter
Prupe.1G189900	Pp01	12352148	12357396	Sugar transporter
Prupe.7G186000	Pp01	12361223	12363662	Sugar transporter
Prupe.4G008400	Pp01	12364066	12366369	Sugar transporter
Prupe.1G157000	Pp01	12380623	12383155	Sugar transporter
Prupe.2G001700	Pp01	12421965	12424261	Sugar transporter
Prupe.2G315500	Pp01	12425073	12427509	Sugar transporter
Prupe.3G034900	Pp01	12439287	12442722	Sugar transporter
Prupe.1G514700	Pp01	12447680	12450686	Sugar transporter
Prupe.1G159700	Pp01	12702147	12709396	SPS
Prupe.1G178400	Pp01	15304141	15307207	Invertase inhibitor
Prupe.8G248600	Pp01	17192664	17203398	Sugar transporter
Prupe.8G052700	Pp01	17553687	17560142	Sugar transporter
Prupe.1G196700	Pp01	18768317	18771430	FK
Prupe.7G148900	Pp01	18768317	18771430	Sugar transporter
Prupe.7G236300	Pp01	23533967	23536346	Sugar transporter
Prupe.1G222100	Pp01	23632798	23636823	G6PDH
Prupe.7G236300	Pp01	25769320	25772751	Sugar transporter
Prupe.1G249100	Pp01	26159090	26159866	Invertase inhibitor
Prupe.1G271500	Pp01	27843811	27849253	Sucrose transporter
Prupe.7G253400	Pp01	27843811	27849253	Sugar transporter
Prupe.1G329200	Pp01	31268448	31271163	PGM

Prupe.1G330700	Pp01	31353882	31359327	PGM
Prupe.1G330800	Pp01	31360037	31362451	Invertase inhibitor
Prupe.1G330900	Pp01	31370947	31371869	Invertase inhibitor
Prupe.1G365400	Pp01	33537122	33539832	Invertase
Prupe.1G366000	Pp01	33563784	33567154	HK
Prupe.1G376200	Pp01	34094935	34099233	AGPase
Prupe.1G378500	Pp01	34198014	34202992	Sugar transporter
Prupe.1G380500	Pp01	34311725	34319938	PGI
Prupe.8G101500	Pp01	34383389	34385937	Sugar transporter
Prupe.1G444000	Pp01	37629021	37632743	PFK
Prupe.8G105600	Pp01	40215648	40219569	Sugar transporter
Prupe.1G488700	Pp01	40683210	40693558	UGPase
Prupe.6G059800	Pp01	40683210	40693558	Sugar transporter
Prupe.5G006300	Pp01	41486624	41488426	Sugar transporter
Prupe.1G501300	Pp01	42463956	42470112	Sugar transporter
Prupe.1G528400	Pp01	43236123	43241935	PGI
Prupe.1G529400	Pp01	43300109	43302153	Invertase inhibitor
Prupe.1G529500	Pp01	43306345	43308503	Invertase inhibitor
Prupe.1G542000	Pp01	44273773	44281717	Sucrose transporter
Prupe.1G070900	Pp01	44341545	44347181	Sugar transporter
Prupe.1G556900	Pp01	45464916	45471252	Invertase
Prupe.1G560800	Pp01	45786677	45788261	F16BPase
Prupe.8G105300	Pp01	47452615	47454370	Sugar transporter
Prupe.3G156900	Pp02	254326	261608	Sugar transporter
Prupe.2G024100	Pp02	2399702	2400551	Sugar transporter
Prupe.1G481800	Pp02	3850627	3861624	Sugar transporter
Prupe.5G114600	Pp02	3872019	3883568	Sugar transporter
Prupe.2G042500	Pp02	4646622	4650777	Sugar transporter
Prupe.2G058200	Pp02	7236001	7241012	Invertase inhibitor
Prupe.2G058300	Pp02	7240338	7242234	Invertase inhibitor
Prupe.2G075000	Pp02	11444370	11449689	Invertase
Prupe.2G083900	Pp02	13283820	13288728	Invertase
Prupe.2G099900	Pp02	15585362	15588499	Invertase inhibitor
Prupe.2G100200	Pp02	15598310	15600150	Invertase inhibitor
Prupe.2G100500	Pp02	15624210	15626339	Invertase inhibitor
Prupe.2G107500	Pp02	16489359	16498039	UGPase
Prupe.2G115600	Pp02	17346686	17347455	Invertase inhibitor
Prupe.2G325500	Pp02	17666417	17669913	Sugar transporter
Prupe.2G245600	Pp02	17786619	17790424	Sugar transporter
Prupe.8G224400	Pp02	17795220	17802381	Sugar transporter
Prupe.2G141000	Pp02	19741431	19744411	Invertase inhibitor
Prupe.2G141200	Pp02	19754635	19757309	Invertase inhibitor
Prupe.2G141800	Pp02	19788161	19788802	Invertase inhibitor
Prupe.2G151100	Pp02	20597751	20600680	FK
Prupe.5G006100	Pp02	22325621	22328702	Sugar transporter
Prupe.2G191400	Pp02	23073519	23078158	Invertase

Prupe.2G191500	Pp02	23086423	23089255	Invertase
Prupe.8G100900	Pp02	23645962	23647161	Sugar transporter
Prupe.2G210700	Pp02	24336961	24339341	Invertase inhibitor
Prupe.2G213100	Pp02	24488528	24494820	PGM
Prupe.2G242300	Pp02	26135523	26137574	SuSy
Prupe.4G127600	Pp02	26274645	26275987	Sugar transporter
Prupe.2G245700	Pp02	26276979	26281137	F16BPase
Prupe.2G253500	Pp02	26681048	26687711	UGPase
Prupe.1G144900	Pp02	27381468	27382915	Sugar transporter
Prupe.2G272600	Pp02	27574029	27579251	PFK
Prupe.2G274100	Pp02	27657485	27659539	Sugar transporter
Prupe.8G253500	Pp02	27663409	27665736	Sugar transporter
Prupe.2G277900	Pp02	27860807	27865049	Invertase
Prupe.2G278200	Pp02	27882912	27884981	Invertase inhibitor
Prupe.2G278300	Pp02	27886643	27888606	Invertase inhibitor
Prupe.2G279700	Pp02	27936617	27937883	Invertase inhibitor
Prupe.2G279800	Pp02	27940209	27941321	Invertase inhibitor
Prupe.2G279900	Pp02	27943416	27944548	Invertase inhibitor
Prupe.5G090900	Pp02	28195368	28196655	Sugar transporter
Prupe.2G286900	Pp02	28284289	28289943	PGM
Prupe.2G287100	Pp02	28297724	28301506	FK
Prupe.5G146500	Pp02	28297724	28301506	Sugar transporter
Prupe.2G288800	Pp02	28363713	28365855	SDH
Prupe.1G156300	Pp02	29182160	29184160	Sugar transporter
Prupe.8G017400	Pp02	29228674	29229557	Sugar transporter
Prupe.2G310600	Pp02	29383444	29387138	Invertase inhibitor
Prupe.2G307700	Pp02	30075681	30080804	Sugar transporter
Prupe.1G187700	Pp02	30201632	30205095	Sugar transporter
Prupe.3G003700	Pp03	248619	251553	Invertase inhibitor
Prupe.3G007700	Pp03	471754	472278	Invertase inhibitor
Prupe.3G007800	Pp03	474999	475518	Invertase inhibitor
Prupe.3G007900	Pp03	476936	477537	Invertase inhibitor
Prupe.3G008000	Pp03	477978	478274	Invertase inhibitor
Prupe.3G009500	Pp03	606163	610062	Invertase
Prupe.3G014100	Pp03	1010886	1015717	SuSy
Prupe.3G015000	Pp03	1069857	1077800	UGPase
Prupe.3G027200	Pp03	2015795	2020810	Sugar transporter
Prupe.3G031000	Pp03	2300979	2311223	Invertase inhibitor
Prupe.1G133300	Pp03	2550134	2551429	Sugar transporter
Prupe.3G048300	Pp03	3405921	3409831	Invertase
Prupe.3G056600	Pp03	4017399	4024584	PFK
Prupe.3G057800	Pp03	4096447	4101575	HK
Prupe.6G224200	Pp03	4773175	4781004	Sugar transporter
Prupe.1G155000	Pp03	5103365	5104285	Sugar transporter
Prupe.1G542000	Pp03	6836304	6839968	Sugar transporter
Prupe.3G144600	Pp03	15757313	15764258	Sugar transporter

Prupe.3G146800	Pp03	16038192	16038906	Invertase inhibitor
Prupe.1G038600	Pp03	16048486	16049516	Sugar transporter
Prupe.3G152800	Pp03	16644957	16648119	Sugar transporter
Prupe.2G307800	Pp03	17531732	17535222	Sugar transporter
Prupe.3G160500	Pp03	17922917	17932622	FK
Prupe.1G271500	Pp03	17922917	17932622	Sugar transporter
Prupe.3G192600	Pp03	20466452	20470025	AGPase
Prupe.3G198600	Pp03	20821071	20825286	G6PDH
Prupe.3G211400	Pp03	21629154	21633616	Sugar transporter
Prupe.3G211800	Pp03	21648145	21653335	PFK
Prupe.8G101200	Pp03	25751273	25753667	Sugar transporter
Prupe.3G300000	Pp03	26516741	26517831	PGI
Prupe.3G300200	Pp03	26531676	26537007	G6PDH
Prupe.3G312600	Pp03	27071117	27075186	F26BPase
Prupe.4G001200	Pp04	131826	132365	Invertase inhibitor
Prupe.4G002000	Pp04	164366	168836	PFK
Prupe.7G223700	Pp04	426241	429137	Sugar transporter
Prupe.4G014600	Pp04	671854	676945	PFK
Prupe.4G025200	Pp04	1195495	1198231	Invertase inhibitor
Prupe.5G128400	Pp04	1255928	1258763	Sugar transporter
Prupe.5G125100	Pp04	2015166	2019155	Sugar transporter
Prupe.3G152800	Pp04	2020923	2022326	Sugar transporter
Prupe.4G072300	Pp04	3536606	3538975	Sugar transporter
Prupe.4G073700	Pp04	3599032	3604715	UGPase
Prupe.8G020100	Pp04	4096771	4100129	Sugar transporter
Prupe.4G115400	Pp04	6203523	6211118	UGPase
Prupe.6G331800	Pp04	6982370	6984007	Sugar transporter
Prupe.4G127200	Pp04	6987171	6992153	Sugar transporter
Prupe.1G516200	Pp04	7020285	7023162	Sugar transporter
Prupe.4G136700	Pp04	7583583	7586022	F16BPase
Prupe.4G083700	Pp04	8912151	8916616	Sugar transporter
Prupe.4G008300	Pp04	8922957	8926748	Sugar transporter
Prupe.7G185800	Pp04	12106768	12111636	Sugar transporter
Prupe.4G206400	Pp04	12882369	12883999	Invertase
Prupe.4G240300	Pp04	15817263	15820307	SDH
Prupe.4G256200	Pp04	17960292	17966684	HK
Prupe.7G247800	Pp04	18369144	18376596	Sugar transporter
Prupe.5G001900	Pp05	311315	318463	SPP
Prupe.5G003400	Pp05	464208	470729	PFK
Prupe.1G156700	Pp05	696032	698833	Sugar transporter
Prupe.6G183300	Pp05	717190	722649	Sugar transporter
Prupe.5G024000	Pp05	2681142	2681904	Invertase
Prupe.5G083900	Pp05	3092369	3095364	Sugar transporter
Prupe.5G046300	Pp05	4993486	4998570	PGM
Prupe.5G048600	Pp05	5172883	5173482	Invertase inhibitor
Prupe.5G048900	Pp05	5183213	5183993	Invertase inhibitor

Prupe.5G075600	Pp05	8955357	8959521	Invertase
Prupe.5G076800	Pp05	9091326	9092648	Invertase inhibitor
Prupe.5G076900	Pp05	9094726	9096486	Invertase inhibitor
Prupe.7G185700	Pp05	9648862	9651237	Sugar transporter
Prupe.7G234100	Pp05	9708196	9711080	Sugar transporter
Prupe.2G306600	Pp05	10124114	10126399	Sugar transporter
Prupe.4G197800	Pp05	10161578	10163817	Sugar transporter
Prupe.5G112600	Pp05	11636050	11636571	Invertase inhibitor
Prupe.4G155700	Pp05	11755403	11759771	Sugar transporter
Prupe.5G091100	Pp05	12303785	12306940	Sugar transporter
Prupe.6G187000	Pp05	13449100	13451508	Sugar transporter
Prupe.4G127800	Pp05	13456636	13458837	Sugar transporter
Prupe.5G175500	Pp05	14922045	14924558	Sugar transporter
Prupe.5G189700	Pp05	15581665	15582560	Invertase inhibitor
Prupe.5G234300	Pp05	17778792	17791526	FBPA
Prupe.5G241700	Pp05	18195911	18200676	SuSy
Prupe.1G070800	Pp05	18204020	18206357	Sugar transporter
Prupe.6G006600	Pp06	563185	564153	Invertase
Prupe.6G029500	Pp06	2294425	2296969	FBPA
Prupe.6G053800	Pp06	3770137	3778361	F26BPase
Prupe.2G274200	Pp06	4154356	4158112	Sugar transporter
Prupe.4G037800	Pp06	4844200	4848466	Sugar transporter
Prupe.6G072900	Pp06	4977474	4978432	Invertase
Prupe.6G120100	Pp06	8914315	8915814	PFK
Prupe.6G122600	Pp06	9128385	9135532	Invertase
Prupe.1G457700	Pp06	13074689	13076679	Sugar transporter
Prupe.8G181000	Pp06	19048482	19052660	Sugar transporter
Prupe.6G211400	Pp06	19552855	19556036	Sugar transporter
Prupe.6G196200	Pp06	20389671	20395970	PFK
Prupe.6G197400	Pp06	20457399	20457958	Invertase inhibitor
Prupe.6G212100	Pp06	22040650	22043959	HK
Prupe.6G229900	Pp06	23318823	23320506	Invertase
Prupe.6G267900	Pp06	25563295	25565896	Invertase
Prupe.5G006600	Pp06	25911446	25913106	Sugar transporter
Prupe.1G187700	Pp06	25918556	25920954	Sugar transporter
Prupe.6G291200	Pp06	26864548	26869512	Invertase
Prupe.6G300000	Pp06	27264692	27269055	glucose catabolic process
Prupe.6G307600	Pp06	27709051	27713355	G6PDH
Prupe.6G309200	Pp06	27789478	27790065	Invertase inhibitor
Prupe.6G309300	Pp06	27791010	27791534	Invertase inhibitor
Prupe.6G309800	Pp06	27815656	27819789	Invertase
Prupe.6G314000	Pp06	28061726	28064959	Invertase
Prupe.6G318500	Pp06	28269921	28272171	Invertase inhibitor
Prupe.6G318600	Pp06	28273021	28274929	Invertase inhibitor
Prupe.1G554800	Pp06	28633939	28638942	Sugar transporter
Prupe.6G358900	Pp06	28639579	28644548	Sugar transporter

Prupe.6G355900	Pp06	30204408	30206428	Sugar transporter
Prupe.1G156400	Pp06	30511085	30516493	Sugar transporter
Prupe.7G003700	Pp07	400727	403494	UGPase
Prupe.7G089300	Pp07	12296588	12300677	SPP
Prupe.7G103200	Pp07	13285991	13289044	Invertase
Prupe.7G103300	Pp07	13293841	13296921	Invertase
Prupe.7G103400	Pp07	13299771	13304593	Invertase
Prupe.7G116500	Pp07	14159140	14162122	FBPA
Prupe.7G116600	Pp07	14164351	14167758	FBPA
Prupe.4G127300	Pp07	16096653	16099920	Sugar transporter
Prupe.7G165400	Pp07	16968676	16970735	FBPA
Prupe.8G077700	Pp07	17989443	17991978	Sugar transporter
Prupe.1G220700	Pp07	17993351	17994662	Sugar transporter
Prupe.6G070700	Pp07	17997535	18000948	Sugar transporter
Prupe.7G190300	Pp07	18243290	18246068	Invertase inhibitor
Prupe.7G190400	Pp07	18247144	18249472	Invertase inhibitor
Prupe.7G190500	Pp07	18250054	18252735	Invertase inhibitor
Prupe.7G190700	Pp07	18257407	18260068	Invertase inhibitor
Prupe.7G192300	Pp07	18350215	18356360	SuSy
Prupe.7G192400	Pp07	18357081	18359221	Invertase inhibitor
Prupe.7G193700	Pp07	18416980	18418060	Invertase inhibitor
Prupe.7G216700	Pp07	19574975	19578477	PFK
Prupe.7G218800	Pp07	19677704	19682250	HK
Prupe.8G017500	Pp07	19920028	19923317	Sugar transporter
Prupe.7G231500	Pp07	20281792	20283590	Sugar transporter
Prupe.1G584600	Pp07	20283858	20286176	Sugar transporter
Prupe.5G027100	Pp07	20396555	20400978	Sugar transporter
Prupe.1G144800	Pp07	20518944	20520963	Sugar transporter
Prupe.1G156500	Pp07	21041784	21046678	Sugar transporter
Prupe.7G249900	Pp07	21151882	21157785	SuSy
Prupe.2G274300	Pp07	21354476	21357899	Sugar transporter
Prupe.7G270900	Pp07	22198854	22202036	AGPase
Prupe.8G248700	Pp07	22198854	22202036	Sugar transporter
Prupe.8G000700	Pp08	98079	100368	FK
Prupe.8G003700	Pp08	302873	308259	SPS
Prupe.8G076100	Pp08	1630354	1632521	Sugar transporter
Prupe.4G042700	Pp08	1834704	1842366	Sugar transporter
Prupe.8G028300	Pp08	2566771	2570494	F16BPase
Prupe.8G038100	Pp08	3725038	3725754	Invertase inhibitor
Prupe.8G052700	Pp08	5814548	5818369	Sucrose transporter
Prupe.1G156900	Pp08	5814548	5818369	Sugar transporter
Prupe.8G070500	Pp08	10340602	10343542	F16BPase
Prupe.3G071100	Pp08	11181030	11190565	Sugar transporter
Prupe.1G156600	Pp08	11690795	11697592	Sugar transporter
Prupe.8G085100	Pp08	11812456	11815003	FBPA
Prupe.8G093900	Pp08	12662374	12667895	AGPase

Prupe.8G100700	Pp08	13186354	13188881	Sugar transporter
Prupe.7G231600	Pp08	13225762	13228930	Sugar transporter
Prupe.1G038700	Pp08	13264239	13266782	Sugar transporter
Prupe.3G283400	Pp08	13618253	13620401	Sugar transporter
Prupe.8G105400	Pp08	13645519	13647838	Sugar transporter
Prupe.8G105500	Pp08	13658102	13660508	Sugar transporter
Prupe.8G101000	Pp08	13699428	13701967	Sugar transporter
Prupe.8G142900	Pp08	15994437	15996971	SDH
Prupe.8G143000	Pp08	15999040	16001622	SDH
Prupe.8G159800	Pp08	16872814	16878303	Invertase
Prupe.2G266200	Pp08	17164258	17169245	Sugar transporter
Prupe.8G180600	Pp08	18110291	18114880	Sugar transporter
Prupe.8G052700	Pp08	20532425	20537087	Sugar transporter
Prupe.2G001700	Pp08	21474403	21479245	Sugar transporter
Prupe.7G152100	Pp08	21479443	21483871	Sugar transporter
Prupe.5G146400	Pp08	21697139	21698821	Sugar transporter
Prupe.8G261300	Pp08	22064410	22065375	Invertase inhibitor
Prupe.8G263900	Pp08	22159222	22161846	Invertase inhibitor
Prupe.8G264000	Pp08	22164915	22167229	Invertase inhibitor
Prupe.8G264100	Pp08	22169289	22173300	Invertase inhibitor
Prupe.8G264300	Pp08	22179197	22184773	SuSy
Prupe.8G267500	Pp08	22322926	22325983	Invertase inhibitor

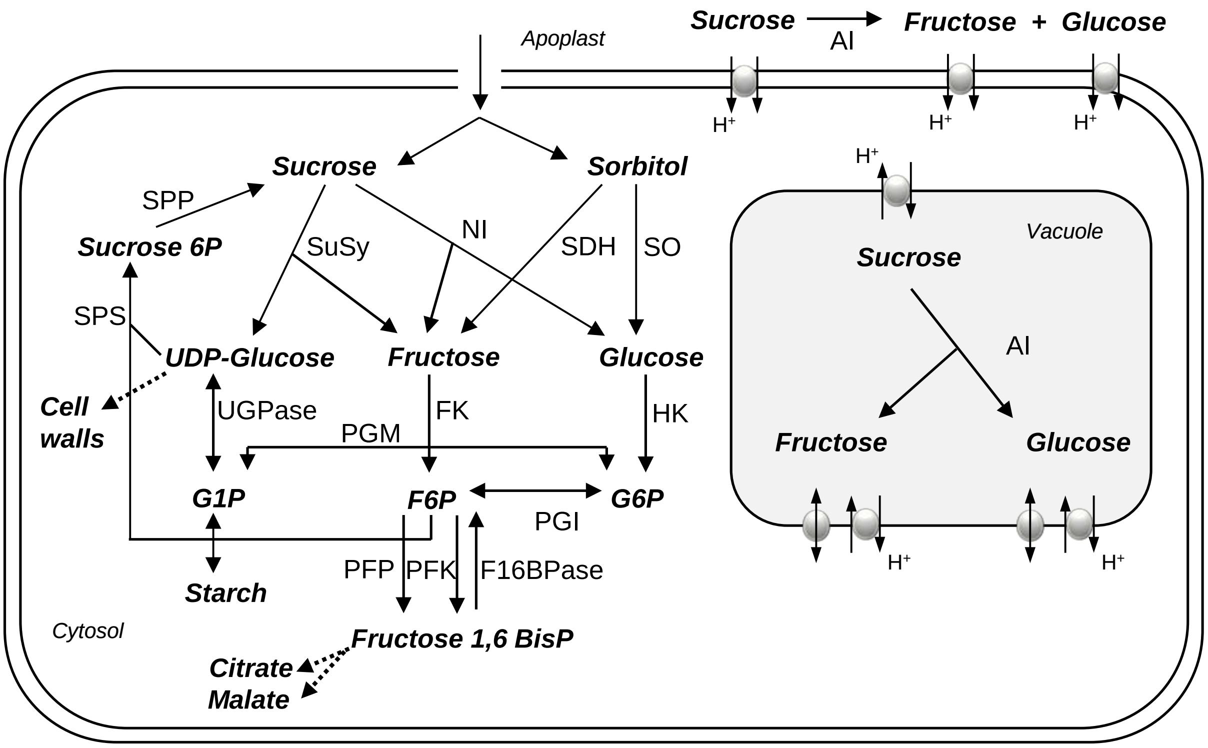

Supplementary Figure S1: Peach sugar metabolism. Abbreviations: acid invertase (AI), fructose-1,6-bisphosphatase (F16BPase), fructose-6-phosphate (F6P), fructokinase (FK), glucose-1-phosphate (G1P), glucose-6-phosphate (G6P), hexokinase (HK), neutral invertase (NI), ATP-phosphofructokinase (PFK), phosphoglucose isomerase (PGI), phosphoglucomutase (PGM), sorbitol dehydrogenase (SDH), PPi-phosphofructokinase (PFP), sorbitol oxidase (SO), sucrose-phosphate phosphatase (SPP), sucrose phosphate synthase (SPS), sucrose (Suc), sucrose synthase (SuSy), and UDP-glucose pyrophosphorylase (UGPase).

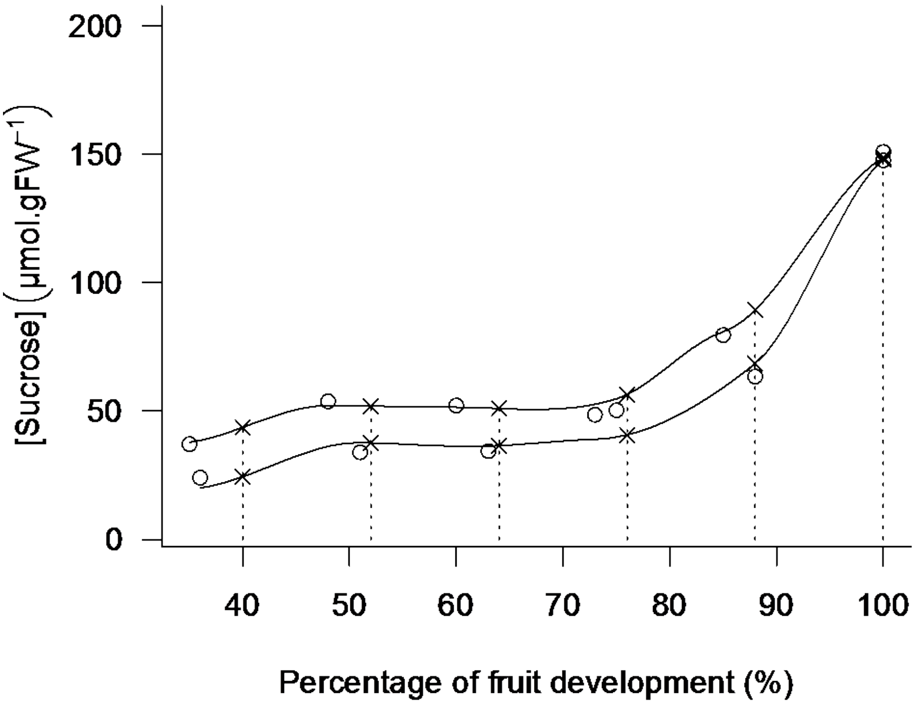

Supplementary Figure S2: Example of rescaling data: sucrose concentration (µmol.gFW-1) during fruit development (in %) for two genotypes. Circles correspond to measured data for the two genotypes and cross correspond to the extracted data used for QTL analyses for the common percentage of fruit development at 40, 52, 64, 76, 88 and 100%.

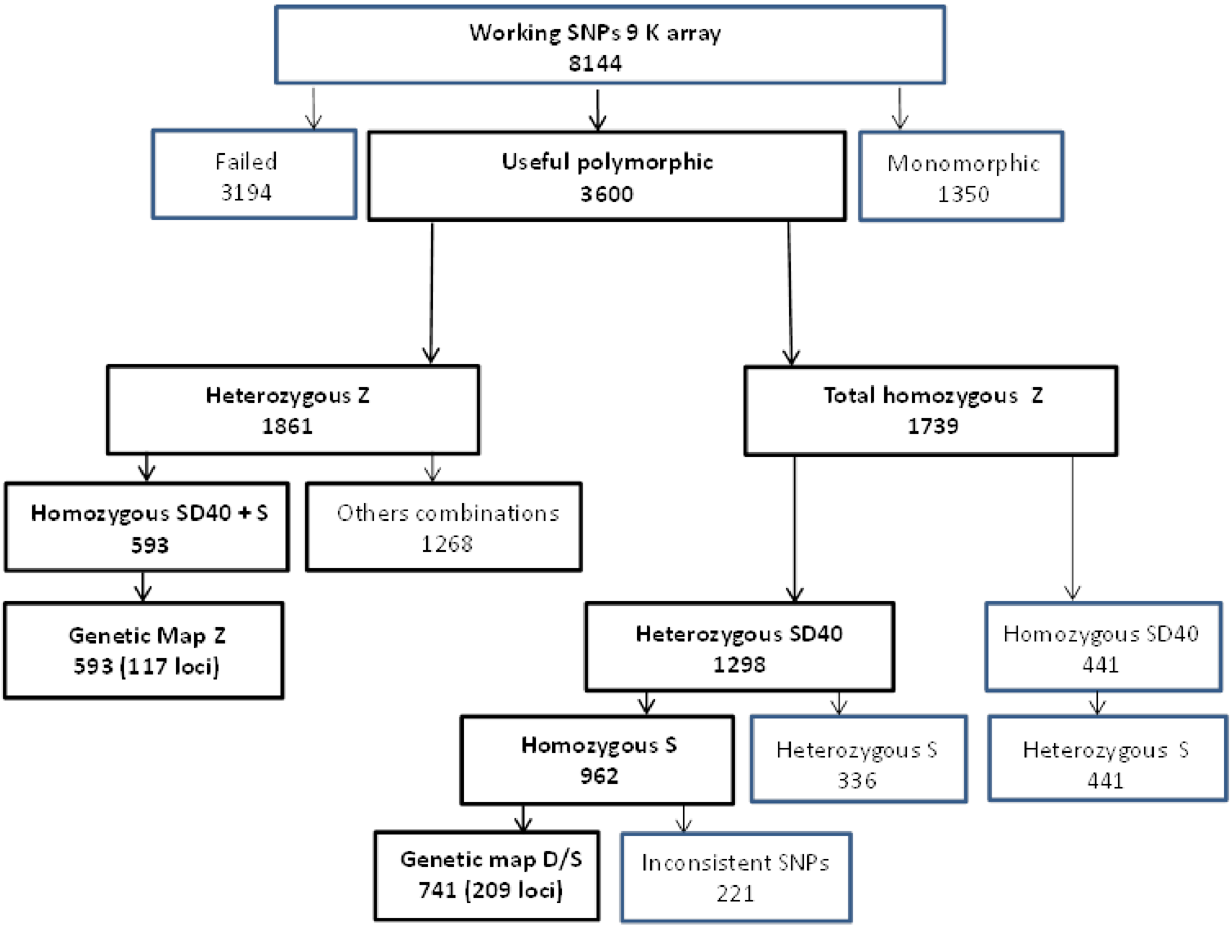

Supplementary Figure S3: Flow chart of the filtering procedure performed to select SNPs that were useful for the construction of the 'DvsS' and the 'SNP_Z' maps. Steps leading to each map are in bold.

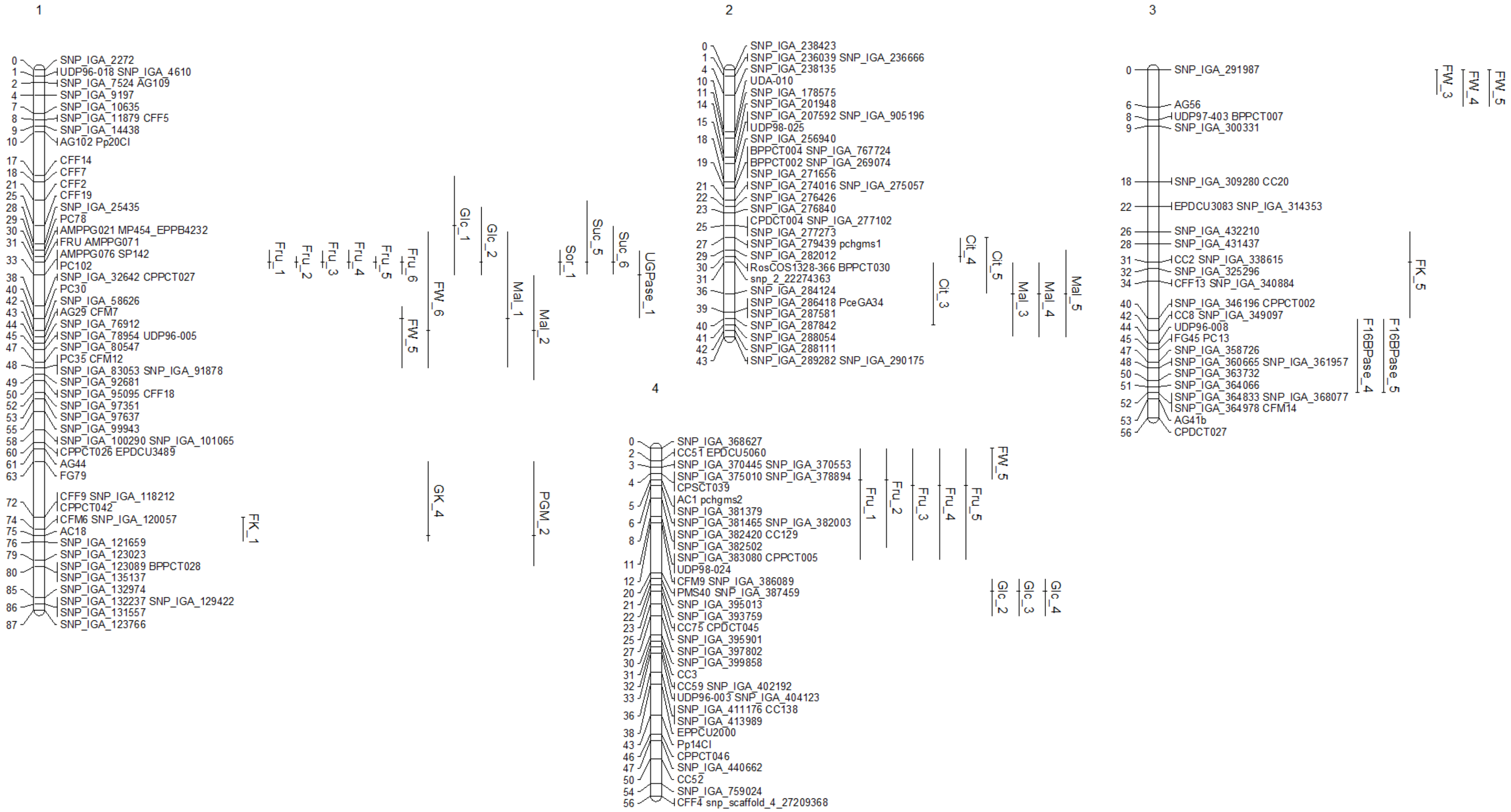
1
0 SNP_IGA_2272
1 UDP96-018 SNP_IGA_4610
2 SNP_IGA_7524 AG109
4 SNP_IGA_9197
7 SNP_IGA_10635
8 SNP_IGA_11879 CFF5
9 SNP_IGA_14438
10 AG102 Pp20CI
17 CFF14
18 CFF7
21 CFF2
25 CFF19
28 SNP_IGA_25435
29 PC78
30 AMPPG021 MP454_EPPB4232
31 FRU AMPPG071
33 AMPPG076 SP142
PC102
38 SNP_IGA_32642 CPPCT027
40 PC30
42 SNP_IGA_58626
43 AG29 CFM7
44 SNP_IGA_76912
45 SNP_IGA_78954 UDP96-005
47 SNP_IGA_80547
48 PC35 CFM12
SNP_IGA_83053 SNP_IGA_91878
49 SNP_IGA_92681
50 SNP_IGA_95095 CFF18
52 SNP_IGA_97351
53 SNP_IGA_97637
55 SNP_IGA_99943
58 SNP_IGA_100290 SNP_IGA_101065
60 CPPCT026 EPDCU3489
61 AG44
63 FG79
72 CFF9 SNP_IGA_118212
CPPCT042
74 CFM6 SNP_IGA_120057
75 AC18
76 SNP_IGA_121659
79 SNP_IGA_123023
80 SNP_IGA_123089 BPPCT028
SNP_IGA_135137
85 SNP_IGA_132974
86 SNP_IGA_132237 SNP_IGA_129422
SNP_IGA_131557
87 SNP_IGA_123766
Fru_1
Fru_2
Fru_3
Fru_4
Fru_5
Fru_6
Glc_1
Glc_2
FW_6
FW_5
Mal_1
Mal_2
Sor_1
Suc_5
Suc_6
UGPase_1
GK_4
PGM_2
FK_1
2
0 SNP_IGA_238423
1 SNP_IGA_236039 SNP_IGA_236666
4 SNP_IGA_238135
10 UDA-010
11 SNP_IGA_178575
14 SNP_IGA_201948
15 SNP_IGA_207592 SNP_IGA_905196
UDP98-025
18 SNP_IGA_256940
19 BPPCT004 SNP_IGA_767724
BPPCT002 SNP_IGA_269074
SNP_IGA_271656
21 SNP_IGA_274016 SNP_IGA_275057
22 SNP_IGA_276426
23 SNP_IGA_276840
25 CPDCT004 SNP_IGA_277102
SNP_IGA_277273
27 SNP_IGA_279439 pchgms1
29 SNP_IGA_282012
30 RosCOS1328-366 BPPCT030
31 snp_2_22274363
36 SNP_IGA_284124
39 SNP_IGA_286418 PceGA34
SNP_IGA_287581
40 SNP_IGA_287842
41 SNP_IGA_288054
42 SNP_IGA_288111
43 SNP_IGA_289282 SNP_IGA_290175
Cit_4
Cit_5
Cit_3
Mal_3
Mal_4
Mal_5
3
0 SNP_IGA_291987
6 AG56
8 UDP97-403 BPPCT007
9 SNP_IGA_300331
18 SNP_IGA_309280 CC20
22 EPDCU3083 SNP_IGA_314353
26 SNP_IGA_432210
28 SNP_IGA_431437
31 CC2 SNP_IGA_338615
32 SNP_IGA_325296
34 CFF13 SNP_IGA_340884
40 SNP_IGA_346196 CPPCT002
42 CC8 SNP_IGA_349097
44 UDP96-008
45 FG45 PC13
47 SNP_IGA_358726
48 SNP_IGA_360665 SNP_IGA_361957
50 SNP_IGA_363732
51 SNP_IGA_364066
52 SNP_IGA_364833 SNP_IGA_368077
SNP_IGA_364978 CFM14
53 AG41b
56 CPDCT027
FW_3
FW_4
FW_5
FK_5
F16BPase_4
F16BPase_5
4
0 SNP_IGA_368627
2 CC51 EPDCU5060
3 SNP_IGA_370445 SNP_IGA_370553
4 SNP_IGA_375010 SNP_IGA_378894
CPSCT039
AC1 pchgms2
5 SNP_IGA_381379
6 SNP_IGA_381465 SNP_IGA_382003
8 SNP_IGA_382420 CC129
SNP_IGA_382502
11 SNP_IGA_383080 CPPCT005
UDP98-024
12 CFM9 SNP_IGA_386089
20 PMS40 SNP_IGA_387459
21 SNP_IGA_395013
22 SNP_IGA_393759
23 CC75 CPDCT045
25 SNP_IGA_395901
27 SNP_IGA_397802
30 SNP_IGA_399858
31 CC3
32 CC59 SNP_IGA_402192
33 UDP96-003 SNP_IGA_404123
36 SNP_IGA_411176 CC138
SNP_IGA_413989
38 EPPCU2000
43 Pp14CI
46 CPPCT046
47 SNP_IGA_440662
50 CC52
54 SNP_IGA_759024
56 CFF4 snp_scaffold_4_27209368
Fru_1
Fru_2
Fru_3
Fru_4
Fru_5
FW_5
Glc_2
Glc_3
Glc_4

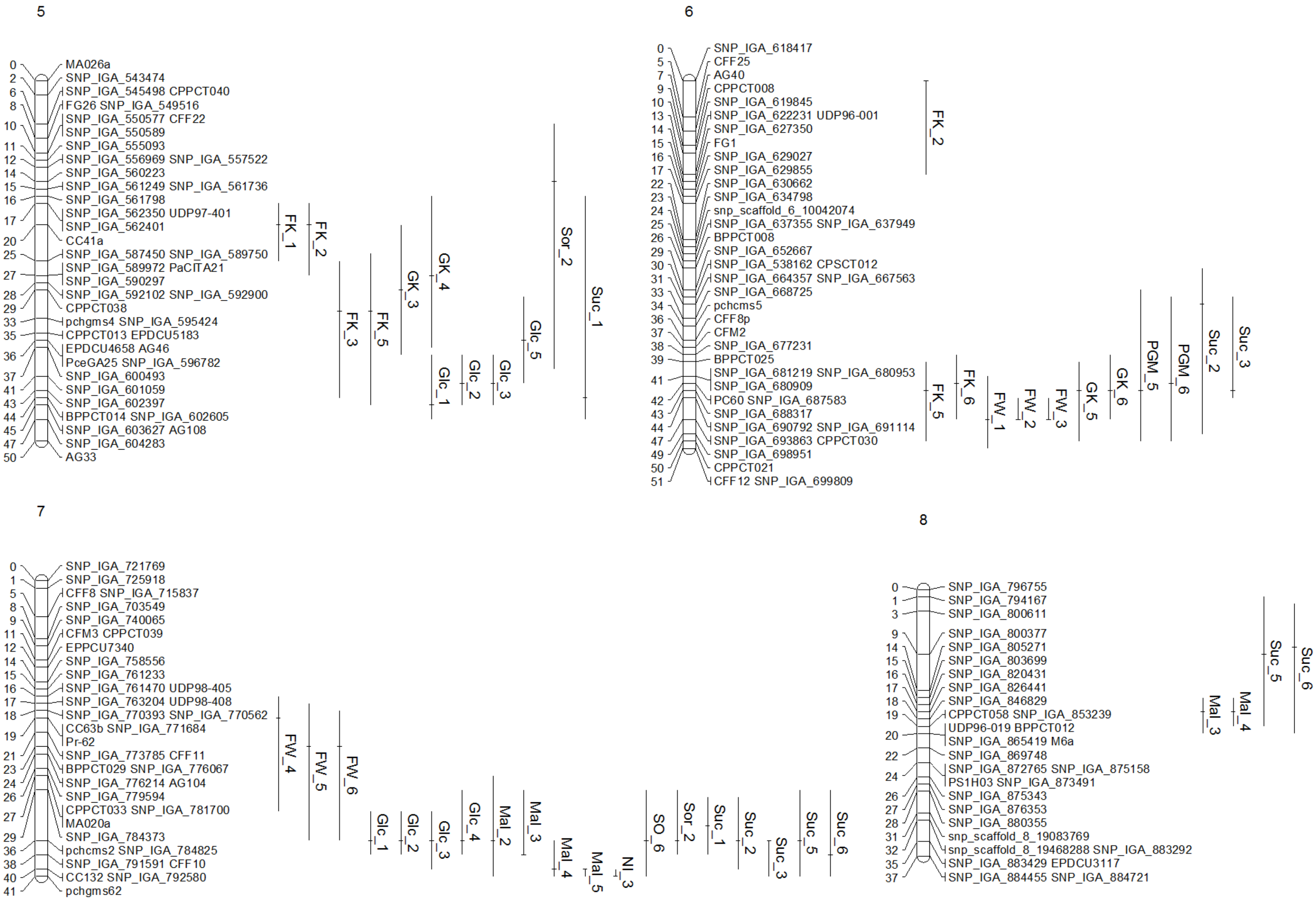
5
0 MA026a
2 SNP_IGA_543474
6 SNP_IGA_545498 CPPCT040
8 FG26 SNP_IGA_549516
10 SNP_IGA_550577 CFF22
SNP_IGA_550589
11 SNP_IGA_555093
12 SNP_IGA_556969 SNP_IGA_557522
14 SNP_IGA_560223
15 SNP_IGA_561249 SNP_IGA_561736
16 SNP_IGA_561798
17 SNP_IGA_562350 UDP97-401
SNP_IGA_562401
20 CC41a
25 SNP_IGA_587450 SNP_IGA_589750
27 SNP_IGA_589972 PaCITA21
SNP_IGA_590297
28 SNP_IGA_592102 SNP_IGA_592900
29 CPPCT038
33 pchgms4 SNP_IGA_595424
35 CPPCT013 EPDCU5183
36 EPDCU4658 AG46
PceGA25 SNP_IGA_596782
37 SNP_IGA_600493
41 SNP_IGA_601059
43 SNP_IGA_602397
44 BPPCT014 SNP_IGA_602605
45 SNP_IGA_603627 AG108
47 SNP_IGA_604283
50 AG33
FK_1
FK_2
FK_3
FK_5
GK_3
GK_4
Glc_1
Glc_2
Glc_3
Glc_5
Sor_2
Suc_1
6
0 SNP_IGA_618417
5 CFF25
7 AG40
9 CPPCT008
10 SNP_IGA_619845
13 SNP_IGA_622231 UDP96-001
14 SNP_IGA_627350
15 FG1
16 SNP_IGA_629027
17 SNP_IGA_629855
22 SNP_IGA_630662
23 SNP_IGA_634798
24 snp_scaffold_6_10042074
25 SNP_IGA_637355 SNP_IGA_637949
26 BPPCT008
29 SNP_IGA_652667
30 SNP_IGA_538162 CPSCT012
31 SNP_IGA_664357 SNP_IGA_667563
33 SNP_IGA_668725
34 pchcms5
36 CFF8p
37 CFM2
38 SNP_IGA_677231
39 BPPCT025
41 SNP_IGA_681219 SNP_IGA_680953
SNP_IGA_680909
42 PC60 SNP_IGA_687583
43 SNP_IGA_688317
44 SNP_IGA_690792 SNP_IGA_691114
47 SNP_IGA_693863 CPPCT030
49 SNP_IGA_698951
50 CPPCT021
51 CFF12 SNP_IGA_699809
FK_2
FK_5
FK_6
FW_1
FW_2
FW_3
GK_5
GK_6
PGM_5
PGM_6
Suc_2
Suc_3
7
0 SNP_IGA_721769
1 SNP_IGA_725918
5 CFF8 SNP_IGA_715837
8 SNP_IGA_703549
9 SNP_IGA_740065
11 CFM3 CPPCT039
12 EPPCU7340
14 SNP_IGA_758556
15 SNP_IGA_761233
16 SNP_IGA_761470 UDP98-405
17 SNP_IGA_763204 UDP98-408
18 SNP_IGA_770393 SNP_IGA_770562
19 CC63b SNP_IGA_771684
Pr-62
21 SNP_IGA_773785 CFF11
23 BPPCT029 SNP_IGA_776067
24 SNP_IGA_776214 AG104
26 SNP_IGA_779594
27 CPPCT033 SNP_IGA_781700
MA020a
29 SNP_IGA_784373
36 pchcms2 SNP_IGA_784825
38 SNP_IGA_791591 CFF10
40 CC132 SNP_IGA_792580
41 pchgms62
FW_4
FW_5
FW_6
Glc_1
Glc_2
Glc_3
Glc_4
Mal_2
Mal_3
Mal_4
Mal_5
NI_3
SO_6
Sor_2
Suc_1
Suc_2
Suc_3
Suc_5
Suc_6
8
0 SNP_IGA_796755
1 SNP_IGA_794167
3 SNP_IGA_800611
9 SNP_IGA_800377
14 SNP_IGA_805271
15 SNP_IGA_803699
16 SNP_IGA_820431
17 SNP_IGA_826441
18 SNP_IGA_846829
19 CPPCT058 SNP_IGA_853239
20 UDP96-019 BPPCT012
SNP_IGA_865419 M6a
22 SNP_IGA_869748
24 SNP_IGA_872765 SNP_IGA_875158
PS1H03 SNP_IGA_873491
26 SNP_IGA_875343
27 SNP_IGA_876353
28 SNP_IGA_880355
31 snp_scaffold_8_19083769
32 snp_scaffold_8_19468288 SNP_IGA_883292
35 SNP_IGA_883429 EPDCU3117
37 SNP_IGA_884455 SNP_IGA_884721
Mal_3
Mal_4
Suc_5
Suc_6

Supplementary Figure S4: Location on the genetic map 'DvsS' (cM) of the QTLs related to the 19 traits studied. Abbreviations: acid invertase (AI), citrate (Cit), fructose-1,6-bisphosphatase (F16BPase), fructokinase (FK), fructose (Fru), fresh weight (FW), glucose (Glc), hexokinase (HK), malate (Mal), neutral invertase (NI), ATP-phosphofructokinase (PFK), phosphoglucomutase (PGM), sorbitol dehydrogenase (SDH), sorbitol oxidase (SO), sucrose phosphate synthase (SPS), sorbitol (Sor), sucrose (Suc), sucrose synthase (SuSy), and UDP-glucose pyrophosphorylase (UGPase). The numbers following the trait names refer to the stages at which the QTL was detected (1 refers to 40% of development, 2 to 52%, 3 to 64%, 4 to 76%, 5 to 88% and 6 to 100%).

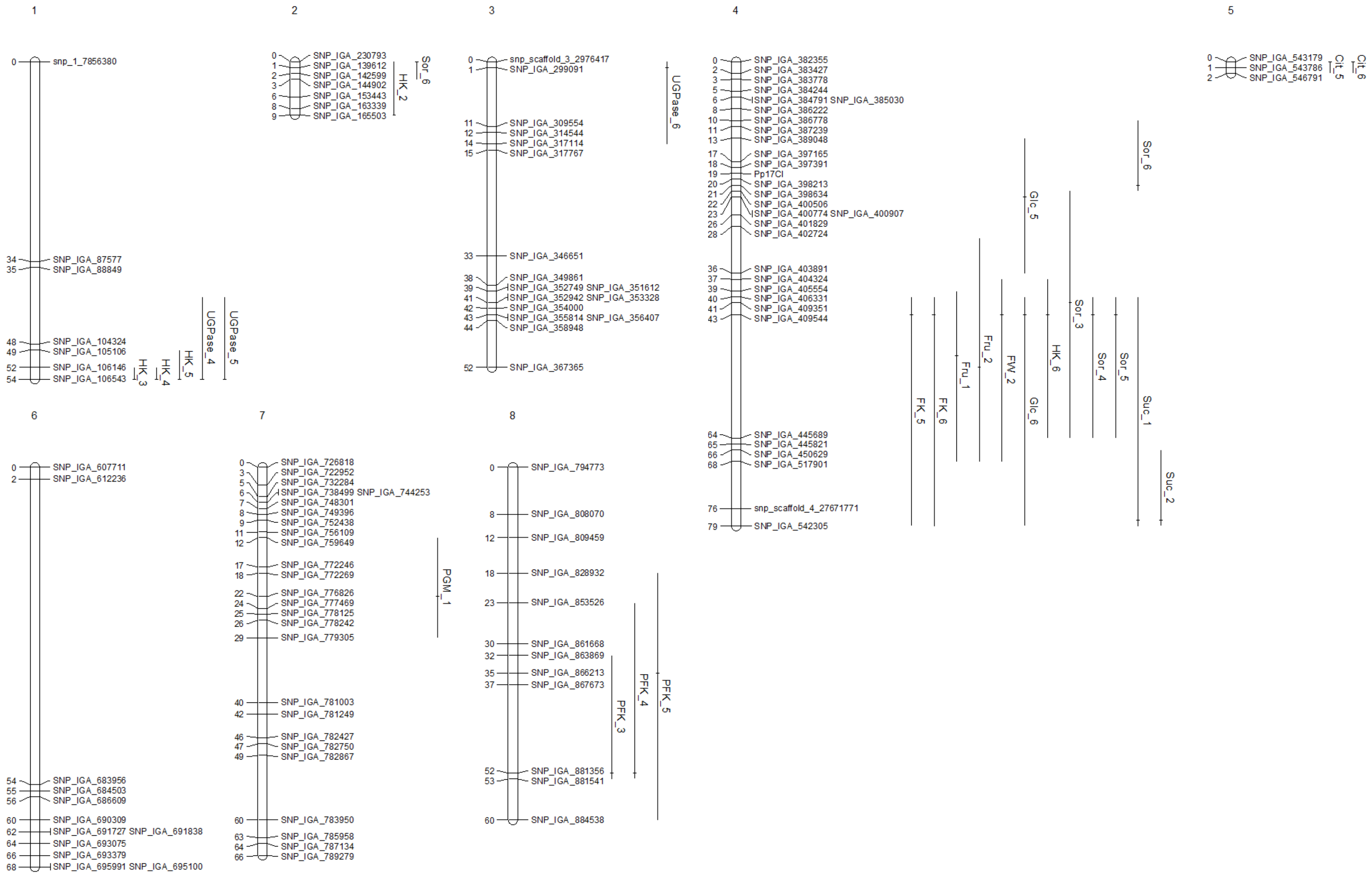
1
0 snp_1_7856380
34 SNP_IGA_87577
35 SNP_IGA_88849
48 SNP_IGA_104324
49 SNP_IGA_105106
52 SNP_IGA_106146
54 SNP_IGA_106543
HK_3
HK_4
HK_5
UGPase_4
UGPase_5
2
0 SNP_IGA_230793
1 SNP_IGA_139612
2 SNP_IGA_142599
3 SNP_IGA_144902
6 SNP_IGA_153443
8 SNP_IGA_163339
9 SNP_IGA_165503
HK_2
Sor_6
3
0 snp_scaffold_3_2976417
1 SNP_IGA_299091
11 SNP_IGA_309554
12 SNP_IGA_314544
14 SNP_IGA_317114
15 SNP_IGA_317767
33 SNP_IGA_346651
38 SNP_IGA_349861
39 SNP_IGA_352749 SNP_IGA_351612
41 SNP_IGA_352942 SNP_IGA_353328
42 SNP_IGA_354000
43 SNP_IGA_355814 SNP_IGA_356407
44 SNP_IGA_358948
52 SNP_IGA_367365
UGPase_6
4
0 SNP_IGA_382355
2 SNP_IGA_383427
3 SNP_IGA_383778
5 SNP_IGA_384244
6 SNP_IGA_384791 SNP_IGA_385030
8 SNP_IGA_386222
10 SNP_IGA_386778
11 SNP_IGA_387239
13 SNP_IGA_389048
17 SNP_IGA_397165
18 SNP_IGA_397391
19 Pp17CI
20 SNP_IGA_398213
21 SNP_IGA_398634
22 SNP_IGA_400506
23 SNP_IGA_400774 SNP_IGA_400907
26 SNP_IGA_401829
28 SNP_IGA_402724
36 SNP_IGA_403891
37 SNP_IGA_404324
39 SNP_IGA_405554
40 SNP_IGA_406331
41 SNP_IGA_409351
43 SNP_IGA_409544
64 SNP_IGA_445689
65 SNP_IGA_445821
66 SNP_IGA_450629
68 SNP_IGA_517901
76 snp_scaffold_4_27671771
79 SNP_IGA_542305
FK_5
FK_6
Fru_1
Fru_2
FW_2
Glc_5
Glc_6
HK_6
Sor_3
Sor_4
Sor_5
Sor_6
Suc_1
Suc_2
5
0 SNP_IGA_543179
1 SNP_IGA_543786
2 SNP_IGA_546791
Cit_5
Cit_6
6
0 SNP_IGA_607711
2 SNP_IGA_612236
54 SNP_IGA_683956
55 SNP_IGA_684503
56 SNP_IGA_686609
60 SNP_IGA_690309
62 SNP_IGA_691727 SNP_IGA_691838
64 SNP_IGA_693075
66 SNP_IGA_693379
68 SNP_IGA_695991 SNP_IGA_695100
7
0 SNP_IGA_726818
3 SNP_IGA_722952
5 SNP_IGA_732284
6 SNP_IGA_738499 SNP_IGA_744253
7 SNP_IGA_748301
8 SNP_IGA_749396
9 SNP_IGA_752438
11 SNP_IGA_756109
12 SNP_IGA_759649
17 SNP_IGA_772246
18 SNP_IGA_772269
22 SNP_IGA_776826
24 SNP_IGA_777469
25 SNP_IGA_778125
26 SNP_IGA_778242
29 SNP_IGA_779305
40 SNP_IGA_781003
42 SNP_IGA_781249
46 SNP_IGA_782427
47 SNP_IGA_782750
49 SNP_IGA_782867
60 SNP_IGA_783950
63 SNP_IGA_785958
64 SNP_IGA_787134
66 SNP_IGA_789279
PGM_1
8
0 SNP_IGA_794773
8 SNP_IGA_808070
12 SNP_IGA_809459
18 SNP_IGA_828932
23 SNP_IGA_853526
30 SNP_IGA_861668
32 SNP_IGA_863869
35 SNP_IGA_866213
37 SNP_IGA_867673
52 SNP_IGA_881356
53 SNP_IGA_881541
60 SNP_IGA_884538
PFK_3
PFK_4
PFK_5

Supplementary Figure S5: Location on the genetic 'SNP_Z' map (cM) of the QTLs related to the 19 traits studied. Abbreviations: acid invertase (AI), citrate (Cit), fructose-1,6-bisphosphatase (F16BPase), fructokinase (FK), fructose (Fru), fresh weight (FW), glucose (Glc), hexokinase (HK), malate (Mal), neutral invertase (NI), ATP-phosphofructokinase (PFK), phosphoglucomutase (PGM), sorbitol dehydrogenase (SDH), sorbitol oxidase (SO), sucrose phosphate synthase (SPS), sorbitol (Sor), sucrose (Suc), sucrose synthase (SuSy), and UDP-glucose pyrophosphorylase (UGPase). The numbers following the trait names refer to the stages at which the QTL was detected (1 refers to 40% of development, 2 to 52%, 3 to 64%, 4 to 76%, 5 to 88% and 6 to 100%).

a

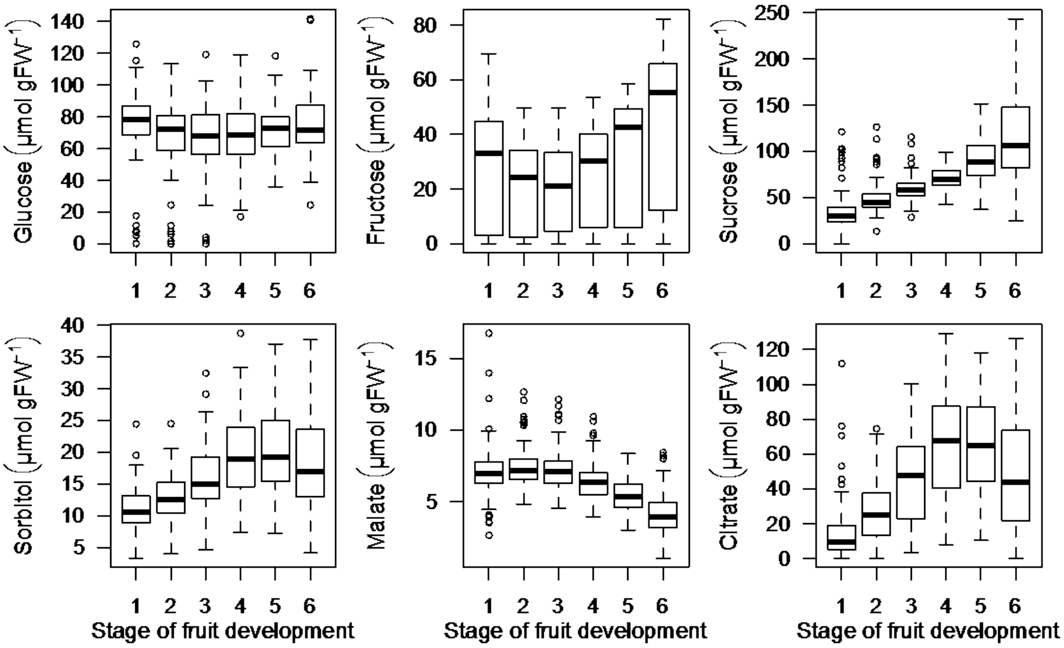

Glucose (µmol gFW⁻¹)
Fructose (µmol gFW⁻¹)
Sucrose (µmol gFW⁻¹)
Sorbitol (µmol gFW⁻¹)
Malate (µmol gFW⁻¹)
Citrate (µmol gFW⁻¹)
Stage of fruit development
Stage of fruit development
Stage of fruit development

b

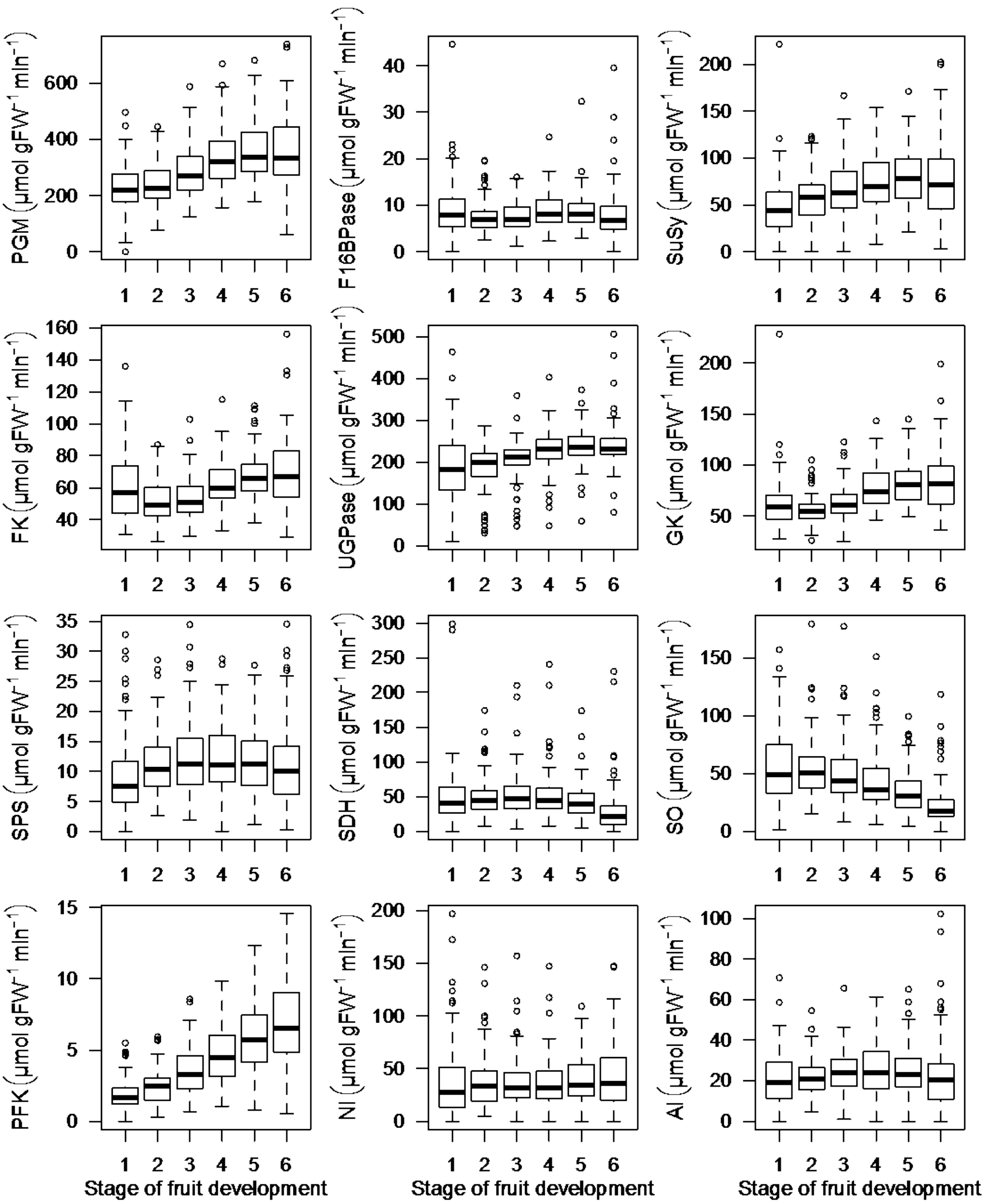
PGM (μmol gFW^{-1} mln^{-1})
F16BPase (μmol gFW^{-1} mln^{-1})
SuSy (μmol gFW^{-1} mln^{-1})
FK (μmol gFW^{-1} mln^{-1})
UGPase (μmol gFW^{-1} mln^{-1})
GK (μmol gFW^{-1} mln^{-1})
SPS (μmol gFW^{-1} mln^{-1})
SDH (μmol gFW^{-1} mln^{-1})
SO (μmol gFW^{-1} mln^{-1})
PFK (μmol gFW^{-1} mln^{-1})
NI (μmol gFW^{-1} mln^{-1})
AI (μmol gFW^{-1} mln^{-1})
Stage of fruit development

c

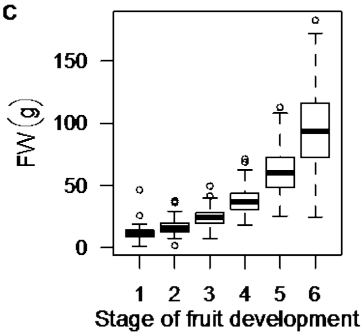
FW (g)
Stage of fruit development

Supplementary Figure S6: Boxplots of (a) sugar and acid concentrations (µmol.gFW^{-1}), (b) enzymatic capacities (µmol.gFW^{-1}min^{-1}) and (c) fresh weight (FW (g)) at the 6 stages of fruit development for all genotypes studied.

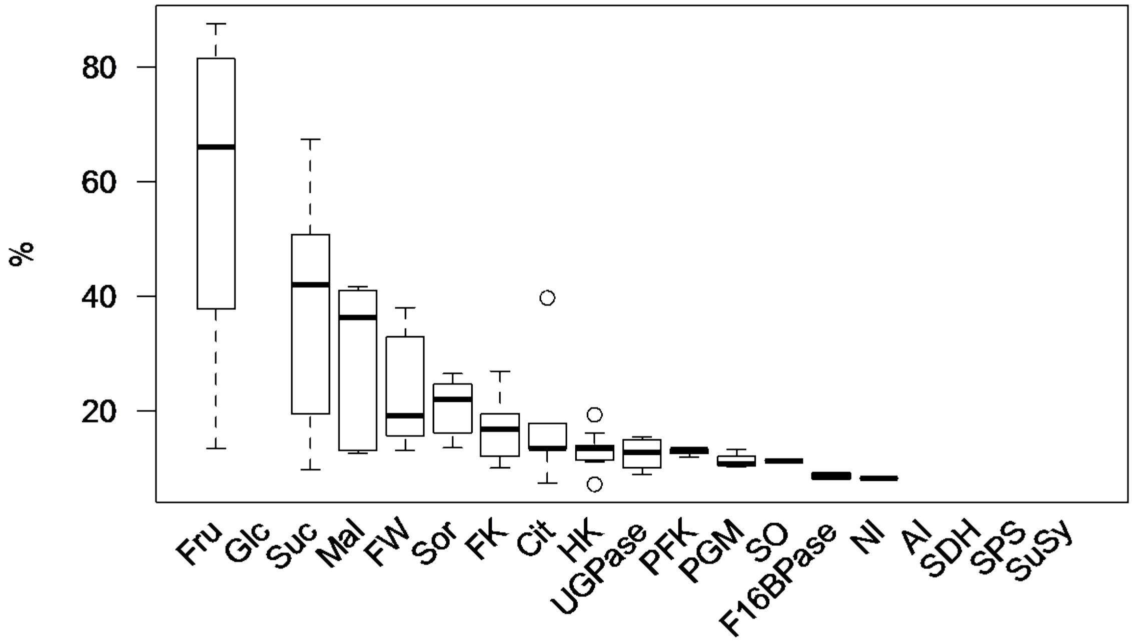

Supplementary Figure S7: Distribution of the total percentage of trait phenotypic variation explained by all of the QTLs detected for a trait. For each trait, the different results come from the different analyses (the six different stages and the two maps). Abbreviations: fructose (Fru), glucose (Glc), sucrose (Suc), malate (Mal), citrate (Cit), sorbitol (Sor), fresh weight (FW), sorbitol dehydrogenase (SDH), fructokinase (FK), UDP-glucose pyrophosphorylase (UGPase), hexokinase (HK), fructose-1,6-bisphosphatase (F16BPase), ATP-phosphofructokinase (PFK), and phosphoglucomutase (PGM), sucrose phosphate synthase (SPS).

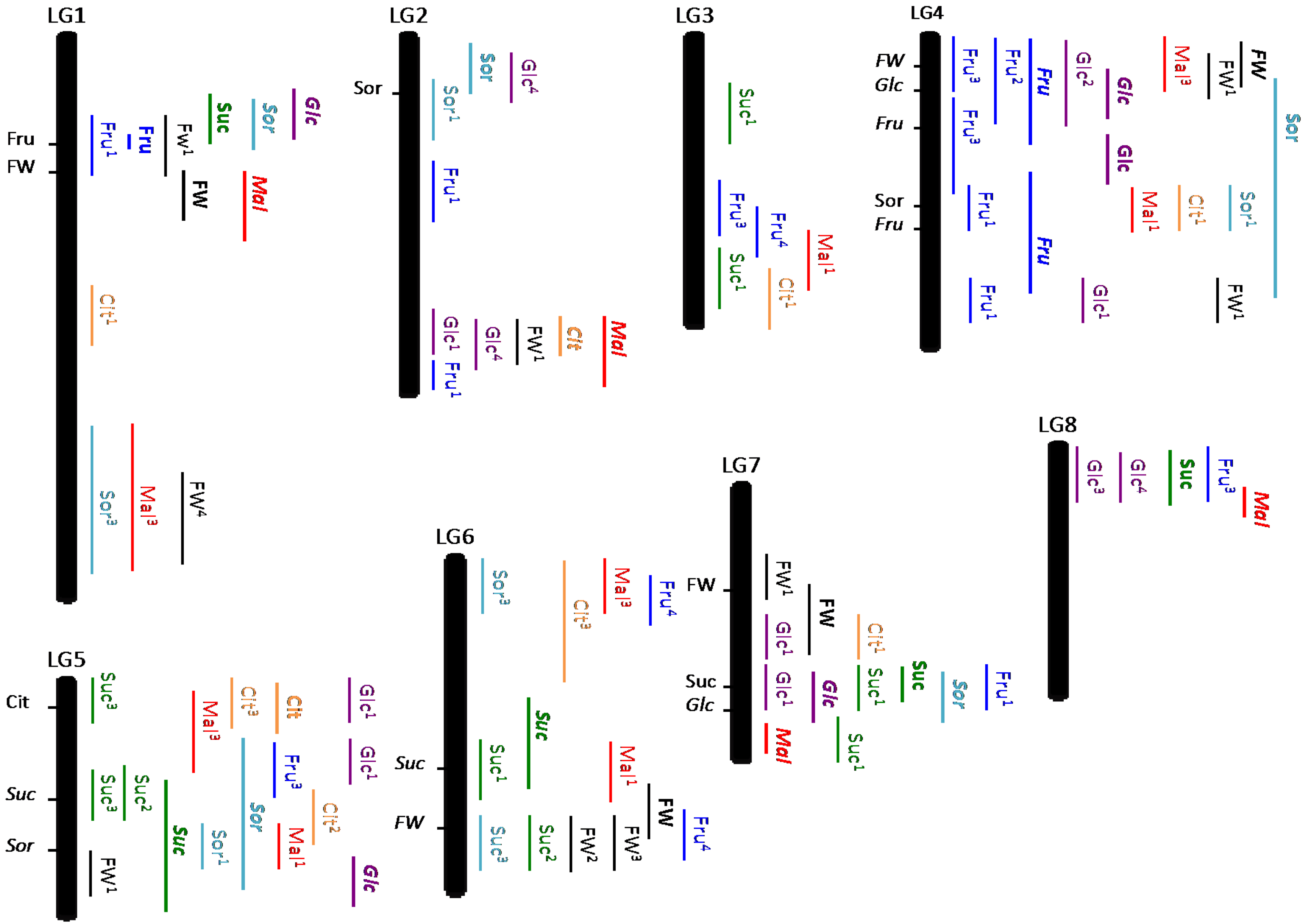

LG1
Fru
FW
Fru1
Fru
Fw1
FW
Suc
Sor
Mal
Glc
Cit1
Sor3
Mal3
FW4
LG2
Sor
Sor
Sor1
Glc4
Fru1
Glc1
Fru1
Glc4
FW1
Cit
Mal
LG3
Suc1
Fru3
Fru4
Suc1
Cit1
Mal1
LG4
FW
Glc
Fru
Sor
Fru
Fru3
Fru3
Fru2
Fru
Glc2
Glc
Glc
Mal3
FW1
FW
Sor
Fru1
Fru
Mal1
Cit1
Sor1
Fru1
Glc1
FW1
LG5
Cit
Suc
Sor
Suc3
Suc3
Suc2
Suc
Mal3
Cit3
Cit
Fru3
Sor
Sor1
Mal1
Cit2
Glc1
Glc1
Glc
FW1
LG6
Suc
FW
Sor3
Cit3
Mal3
Fru4
Suc
Suc1
Mal1
FW
Suc3
Suc2
FW2
FW3
Fru4
LG7
FW
Suc
Glc
FW1
FW
Glc1
Glc1
Glc
Mal
Cit1
Suc1
Suc1
Suc
Sor
Fru1
LG8
Glc3
Glc4
Suc
Fru3
Mal

Supplementary Figure S8: Schematic representation of QTLs for sugar, acid and fresh weight from the literature reported together with QTLs from the present study. QTLs from the present study are represented in bold and QTLs that are not detected at maturity are represented in italics. Co-locations between QTLs detected in this study and in previous studies are indicated in the left side of the linkage group (LG). Abbreviations: fructose (Fru), glucose (Glc), sucrose (Suc), malate (Mal), citrate (Cit), sorbitol (Sor), and fresh weight (FW). [1] (Quilot *et al*. 2004), [2] (Etienne *et al*.2002), [3] (Dirlewanger *et al*. 1999), [4] (Sosinki *et al*. 1998). The population studied in [1] (Quilot *et al*. 2004) is the same as that used in the present study.
